# Nb-induced stabilisation of p53 in HPV-infected cells

**DOI:** 10.1038/s41598-019-49061-9

**Published:** 2019-09-03

**Authors:** Anneleen Steels, Laura Vannevel, Olivier Zwaenepoel, Jan Gettemans

**Affiliations:** Department of Biomolecular Medicine, Faculty of Medicine and Health Sciences, Campus Rommelaere, A. Baertsoenkaai 3, Ghent University, Ghent, Belgium

**Keywords:** Tumour virus infections, Tumour-suppressor proteins

## Abstract

Cervical cancer is caused by a persistent infection of the mucosal epithelia with high-risk human papilloma viruses (HPVs). The viral oncoprotein E6 is responsible for the inactivation of the tumour suppressor p53 and thus plays a crucial role in HPV-induced tumorigenesis. The viral E6 protein forms a trimeric complex with the endogenous E3 ubiquitine ligase E6AP and the DNA-binding domain (DBD) of p53, which results in the polyubiquitination and proteasomal degradation of p53. We have developed nanobodies (Nbs) against the DBD of p53, which substantially stabilise p53 in HeLa cells. The observed effect is specific for HPV-infected cells, since similar effects were not seen for U2OS cells. Despite the fact that the stabilised p53 was strongly nuclear enriched, its tumour suppressive functions were hampered. We argue that the absence of a tumour suppressive effect is caused by inhibition of p53 transactivation in both HPV-infected and HPV-negative cells. The inactivation of the transcriptional activity of p53 was associated with an increased cellular proliferation and viability of HeLa cells. In conclusion, we demonstrate that p53 DBD Nbs positively affect protein stability whilst adversely affecting protein function, attesting to their ability to modulate protein properties in a very subtle manner.

## Introduction

Cervical cancer is the fourth most common cancer in woman worldwide with a ratio of mortality to incidence of 52%. The burden of cervical cancer can be reduced by effective screening programs and vaccination strategies, but these are often lacking in developing countries. Consequently, in those regions the disease still takes its toll on young woman which results in 270.000 deaths annually^1,2^. Cervical cancer is caused by a persistent infection of basal epithelial cells with high-risk human papillomavirus (HPV), whereby 71% of all cases are attributable to infection with the high-risk genotypes HPV16 and HPV18^3^. A substantial part of the oncogenic activity of HPV is related to its ability to interfere with the p53 signalling pathway. In general, the tumour suppressor p53 prevents uncontrolled proliferation of transformed cells by inducing cell cycle arrest, apoptosis or senescence^4^. Less known non-canonical functions of p53, like its involvement in metabolism, autophagy and necrosis, also appear to play a role in tumour suppression^5^. The final biological outcome is the result of an intricate interaction between transcription-dependent and –independent functions of activated p53 and may vary according to the cell type and the imposed cellular stressor^6^. The viral, non-enzymatic, proteins E6 and E7 are fundamental for HPV-induced carcinogenesis. Both proteins interfere with pathways involved in cellular transformation or the immune response through the formation of complexes with cellular proteins. Their coordinate action results in the accumulation of DNA damage in the cell which ultimately promotes tumorigenesis. The tumour suppressor proteins p53 and pRb are important targets for these viral proteins. More specifically, E7 stimulates cellular proliferation by the inactivation of pRb. Under normal circumstances, this would trigger the activation of p53 and would result in the elimination of the cell via cell cycle arrest or apoptosis. However, this response is blocked since E6 targets p53 for proteasomal degradation via the formation of a trimeric p53-E6/E6AP complex^7,8^. The structural details of this trimeric complex have recently been resolved. Prior to p53 binding, the viral E6 protein interacts with the endogenous E3 ubiquitin ligase E6AP via recognition of a conserved LxxLL motif. This interaction induces a conformational switch with the formation of a p53-binding cleft on the E6 protein. The DBD of p53 is targeted by this heterodimer, resulting in the polyubiquitination and proteasomal degradation of the tumour suppressor^9^. Previous research has demonstrated the potency of single-domain antibodies, also known as Nbs, to block interactions between proteins^10^. In this regard, we aimed to target the p53-E6/E6AP interaction by means of Nbs that were specifically developed against the DBD of p53. This type of antibody is obtained by isolating the variable heavy chain domain (VHH) of heavy-chain-only antibodies (HCAbs), which can be found in the sera of *Camelidae*. Nbs are excellent tools for targeting intracellular proteins, since they demonstrate a superb intracellular stability and retain their functionality in the reducing intracellular environment. Furthermore, their small size (~15 kDa), prolate shape and long protruding CDR3 loop allows them to target epitopes that are inaccessible for other antibody-formats. In addition, Nbs have a low immunogenicity although humanisation is routinely performed before clinical application^11,12^. In this study, we demonstrate that intracellular expression of p53 DBD Nbs in HPV-infected HeLa cells results in a significant stabilisation of p53 and in an augmentation of p53 half-life. p53 DBD Nb120 had the most profound impact on p53 levels. Similar effects were not seen when the Nbs were expressed in HPV-negative U2OS cells. Since the effect is specific for HPV-infected cells, we believe that it might result from the interference of the Nbs with HPV-induced proteasomal degradation of p53. Next, the functional impact of p53 stabilisation in HeLa cells was analysed. Despite the fact that p53 was strongly nuclear enriched, an increase of its transactivation activity was not observed. By contrast, the Nbs appear to disrupt the transactivation activities of p53, which was associated with a significant improvement of the cellular proliferation and stability of HeLa cells. Given the impact of the p53 DBD Nbs on p53 functionality, it is thus of interest to identify the targeted epitopes in order to unravel the mechanisms behind the inactivation of p53.

## Results

### *In vivo* validation of p53 DBD Nbs

**Figure 1:**
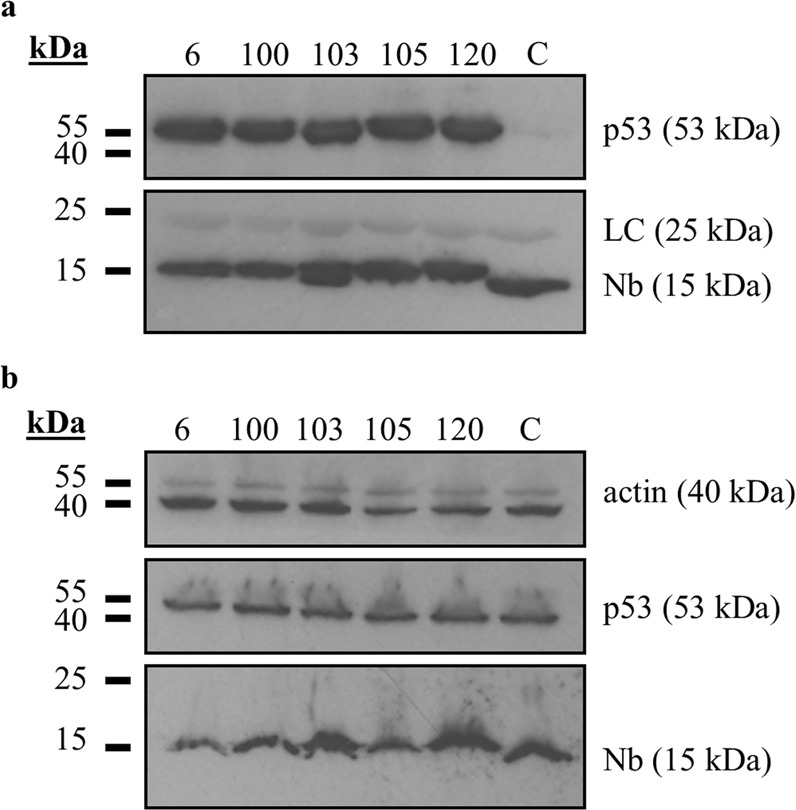
*In vivo* validation of p53 DBD Nbs. (**a)** Pull down of endogenous wild type p53 in HEK293T cells that transiently express FLAG-tagged p53 DBD Nbs. Crude lysates (1 mg) of transfected HEK293T cells were incubated with anti-FLAG M2 affinity gel, resulting in the immobilisation of the FLAG-tagged Nbs. As a negative control, HEK293T cells were transiently transfected with a FLAG-tagged GFP Nb (C). Co-precipitation of endogenous wild type p53 was observed for all p53 DBD Nbs, whilst it was absent for the negative control. **(b)** Expression levels of the transfected FLAG-tagged Nbs in crude lysates of HEK293T cells (40 µg). Nbs were expressed at similar levels. For reasons of clarity and conciseness, blots were cropped to the bands of interest. Full-length blots are depicted in Supplementary Fig. S10. (LC = light chain of IgG antibody).

Nbs against the DBD of p53 were generated by immunisation of an alpaca with recombinant untagged p53 DBD (AA 92-312). Antigen-specific binders were selected through phage-panning. Five Nbs were developed (Nb6, Nb100, Nb103, Nb105 and Nb120). These Nbs were subcloned into the mammalian expression vector pMET7-FLAG and were subsequently evaluated for their potential to bind p53 in the intracellular environment following transfection in HEK293T cells. The *in vivo* pull-down assay revealed that each p53 DBD Nb was capable of co-precipitating endogenous p53, thus confirming their intracellular functionality (Fig. 1a). By contrast, endogenous p53 was not co-precipitated by the negative control which consisted of HEK293T cells that transiently expressed a GFP Nb. The Nbs were expressed at similar levels in HEK293T cells (Fig. 1b).

### p53 DBD Nbs elicit increased p53 levels in HPV-infected cells

Next, we tested if p53 DBD Nbs were capable of enhancing the stability of p53 in HPV-infected cells, since the viral E6 protein and the endogenous ubiquitin protein ligase E6AP target the DBD of p53^9^.

**Figure 2:**
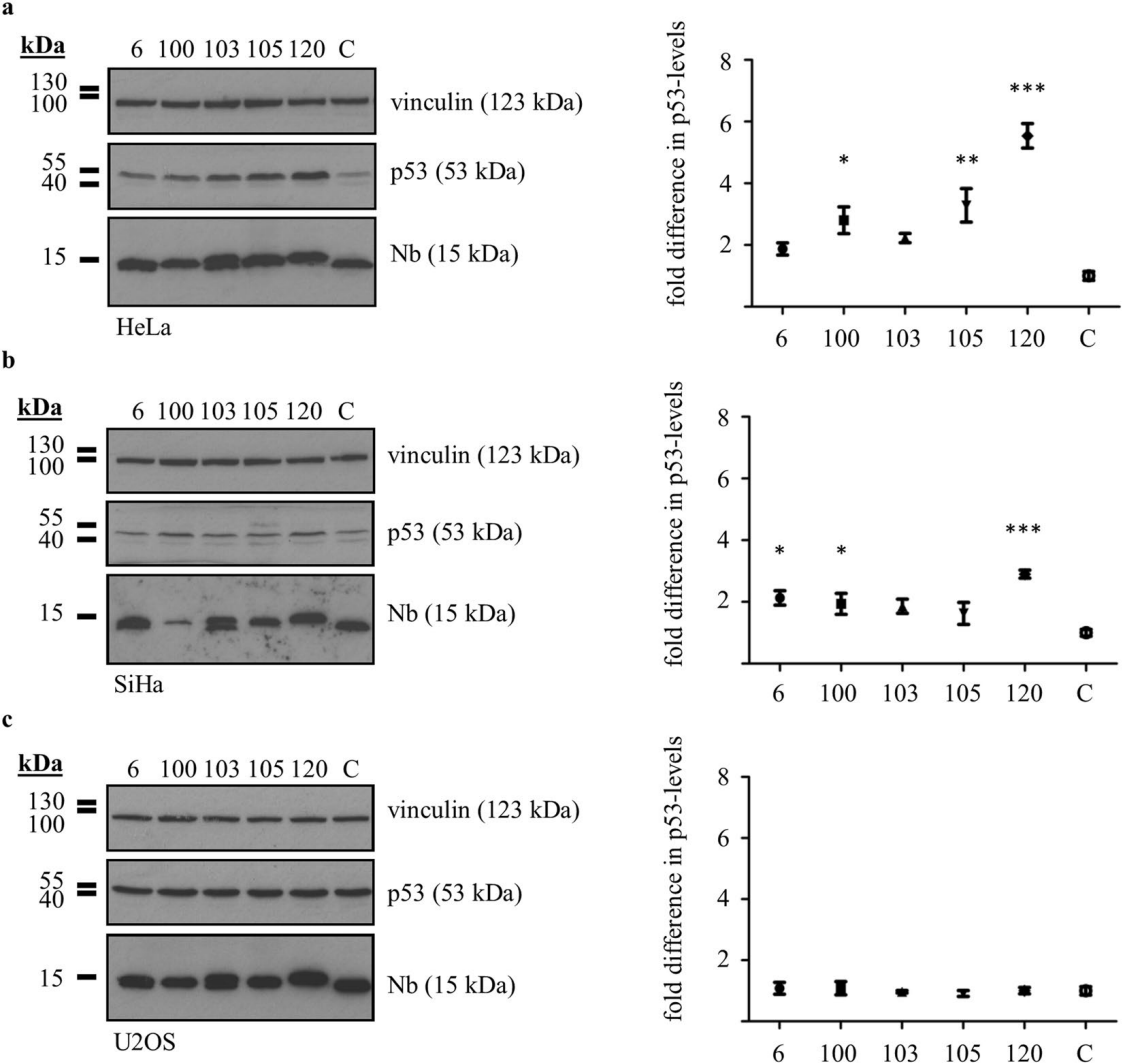
Significant increase of endogenous p53 levels in HPV-infected cells in the presence of p53 DBD Nbs. FLAG-tagged p53 DBD Nbs were transiently expressed in HPV-infected cells (HeLa **(a)** and SiHa **(b)**) or HPV-negative cells (U2OS **(c)**). Crude lysates (60 µg (HeLa and SiHa) or 40 µg (U2OS)) were prepared 24 h after transfection and p53 expression levels were evaluated through western blotting. In the negative control, cells were transfected with a FLAG-tagged GFP Nb (C). Quantitative analysis of protein levels was performed via ImageJ. Vinculin was used as loading control. Values are represented as the mean fold difference in p53 levels (±SEM) relative to the p53 levels measured for the negative control in 4 independent experiments. Statistical analysis was performed via a one-way ANOVA, with Dunnett’s multiple comparison test with a p-value < 0.05. **(a)** p53 levels were significantly increased in HeLa cells in the presence of p53 DBD Nb100 (p < 0,05, 2.8-fold increase of p53 levels), p53 DBD Nb105 (p < 0,01, 3.28-fold increase of p53 levels) and p53 DBD Nb120 (p < 0,001, 5.54-fold increase of p53 levels). **(b)** p53 levels were significantly increased in SiHa cells in the presence of p53 DBD Nb6 (p < 0.05, 2.12-fold increase of p53 levels), p53 DBD Nb100 (p < 0,05, 1.94-fold increase of p53 levels) and p53 DBD Nb120 (p < 0,001, 2.90-fold increase of p53 levels). **(c)** The p53 levels were unaffected by the presence of p53 DBD Nbs in U2OS cells. For reasons of clarity and conciseness, blots were cropped to the bands of interest. Full-length blots are depicted in Supplementary Fig. S10.

To this end, p53 DBD Nbs were transiently expressed in HeLa cells (HPV18) or SiHa cells (HPV16) and crude lysates of the cells were prepared 24 h after transfection. Subsequently, p53 levels were analysed and compared to the negative control where HPV-infected cells expressed an unrelated GFP Nb. Overall, intracellular expression of p53 DBD Nbs resulted in increased p53 levels. Compared to the negative control, significantly higher p53 levels were detected in HeLa cells expressing p53 DBD Nb100 (p < 0.05, 2.8-fold increase of p53 levels), p53 DBD Nb105 (p < 0.01, 3.28-fold increase of p53 levels) or p53 DBD Nb120 (p < 0.001, 5.54-fold increase of p53 levels) (Fig. 2a). In SiHa cells, significant differences were detected in the presence of p53 DBD Nb6 (p < 0.05, 2.12-fold increase of p53-levels), p53 DBD Nb100 (p < 0.05, 1.94-fold increase of p53 levels) and p53 DBD Nb120 (p < 0.001, 2.90-fold increase of p53-levels) (Fig. 2b). Interestingly, similar alterations in p53 levels were not detected when the p53 DBD Nbs were transiently expressed in HPV-negative U2OS cells (Fig. 2c). The experiment was repeated 4 times for each cell line (Supplementary Figs S1 and S2).

Based on these results, p53 DBD Nb120 and p53 DBD Nb105 were selected for further examination of their impact on p53 in HPV-infected cells. The former had the most pronounced influence on p53 levels in both HeLa cells and SiHa cells. The latter was responsible for the second largest increase of p53 levels in HeLa cells, although no significant effect was detected in SiHa cells.

**Figure 3:**
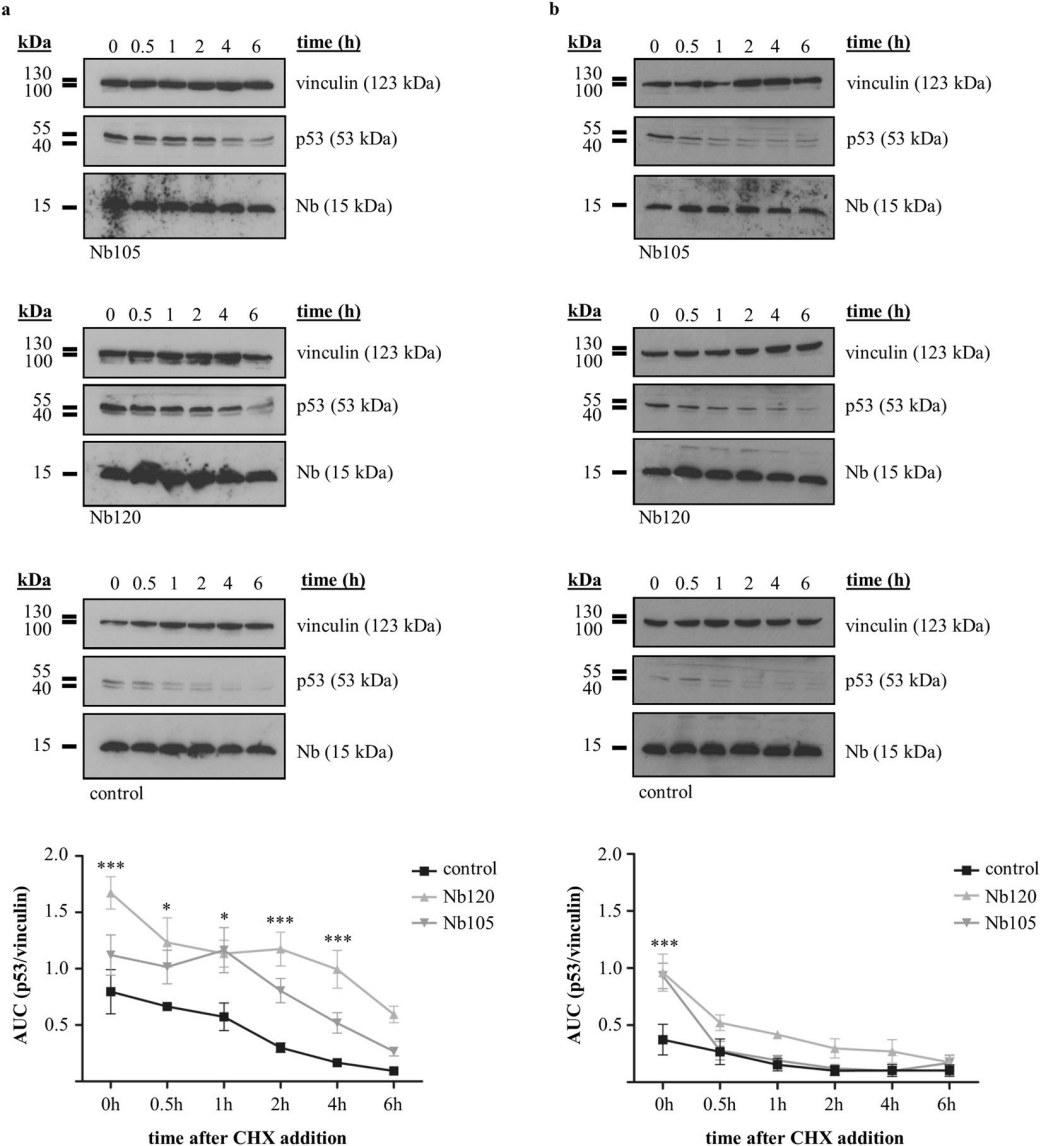
Expression of p53 DBD Nb120 substantially increases p53 protein stability in HeLa cells. A cycloheximide (CHX) pulse chase assay was performed to determine the influence of p53 DBD Nbs on the stability of p53 in HeLa **(a)** and SiHa **(b)** cells. Cells expressing FLAG-tagged p53 DBD Nbs were treated 24 h after transient transfection with 20 µg/ml CHX. Crude lysates (60 µg) were prepared at different time-points after CHX addition (i.e. 0 h, 0.5 h, 1 h, 2 h, 4 h and 6 h) and p53 expression levels were evaluated via western blot. A negative control consisted of cells transfected with an unrelated FLAG-tagged GFP Nb (control). Vinculin served as a loading control and quantitative analysis of protein levels was performed via ImageJ. Values are represented as mean AUC (p53/vinculin) (±SEM) measured in 4 independent experiments. Statistical analysis was performed via a two-way ANOVA, with Bonferroni post-test. **(a)** In HeLa cells expressing Nb120, p53 was stabilised whereby significantly higher p53 levels were detected at t = 0 h (p < 0.001), t = 0.5 h (p < 0.05), t = 1 h (p < 0.05), t = 2 h (p < 0.001) and t = 4 h (p < 0.001). A substantial stabilisation of p53 levels was also observed in cells expressing Nb105, albeit a significant difference was detected only at t = 1 h (p < 0.05). **(b)** A similar stabilisation of p53 was not observed in SiHa cells expressing the p53 DBD Nbs, where a significant increase in p53 levels was detected only at t = 0 (p < 0.001). Nevertheless, when p53 DBD Nb120 was intracellularly expressed, a slight increase in p53 levels was observed for several time points. Blots were cropped to the bands of interest. Full-length blots are depicted in Supplementary Fig. S11. Samples derived from the same experiment and gels/blots were processed in parallel.

A cycloheximide (CHX) pulse chase assay was performed to assess whether the Nbs enhanced the stability of p53 and thus prolonged its half-life. Novel protein synthesis was inhibited 24 h after transfection of the HPV-infected cells by the addition of 20 µg/ml CHX. Crude lysates of the cells were prepared at different time-points (i.e. 0 h, 0.5 h, 1 h, 2 h, 4 h or 6 h) after CHX addition and p53 levels were evaluated. In general, p53 levels were hardly detectable for the negative control in both HeLa cells and SiHa cells (Fig. 3, control). By contrast, p53 levels and p53 half-life in HeLa cells were substantially increased when p53 DBD Nbs were expressed (Fig. 3a and Supplementary Fig. S4). In the presence of p53 DBD Nb120, significantly higher p53 levels were detected up to 4 h after CHX addition (t = 0 h (p < 0.001), t = 0.5 h (p < 0.05), t = 1 h (p < 0.05), t = 2 h (p < 0.001) and t = 4 h (p < 0.001)). Similar observations were made for HeLa cells expressing p53 DBD Nb105, albeit a significant effect was only observed at 1 h after the addition of CHX (p < 0.05) (Fig. 3a). In accordance with these observations, the half-life of p53 increased from ±2 h to ±4 h and 5 h in the presence of p53 DBD Nb105 and p53 DBD Nb120, respectively (Supplementary Fig. S4). In SiHa cells, the p53 DBD Nbs had no impact on p53 half-life. The p53 DBD Nbs elicited a significant augmentation of p53 levels at the start of the experiment (t = 0 (p < 0.001)), but this effect did not persist in time (Fig. 3b). However, even though the result is not significant, in the presence of p53 DBD Nb120, a modest increase in p53 levels was observed at several time points (Fig. 3b). Similar observations were made in repetitive experiments (Supplementary Figs S3 and S5).

**Figure 4:**
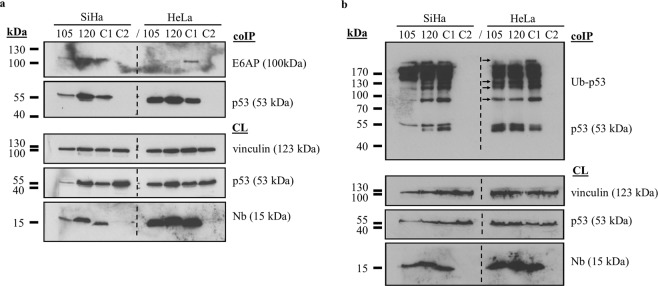
The p53 DBD Nbs appear to hamper the interaction between p53 and E6AP in HeLa cells, which coincides with a small reduction of p53 ubiquitination. Cells transfected with FLAG-tagged p53 DBD Nbs or a FLAG-tagged GFP Nb (C1) were treated 24 h after transfection with 10 µM MG132 (HeLa cells) or 25 µM MG132 (SiHa cells) for a duration of 2 h. p53 was co-immunoprecipitated from the crude lysates (1 mg) whereafter co-precipitation of E6AP (**a**) or ubiquitination of p53 (**b**) was evaluated via western blotting. In order to exclude non-specific binding between p53 and the protein G sepharose beads, an additional negative control was implemented whereby the crude lysate of MG132-treated untransfected cells was incubated with the beads in the absence of the p53-specific antibody (C2). Expression levels of the transfected FLAG-tagged Nbs were evaluated in crude lysates (60 µg) of the cells (CL). (**a**) Equal amounts of E6AP were co-precipitated with p53 in SiHa cells, irrespective of whether or not a p53 DBD Nb was expressed. By contrast, substantially lower amounts of the p53-E6AP complex were detected in HeLa cells expressing a p53 DBD Nb. (**b**) A slight decrease in p53 ubiquitination was detected in HeLa cells when p53 DBD Nbs were intracellularly expressed (indicated by arrows). Differences in p53 ubiquitination in SiHa cells are the result of differences in protein loading. For reasons of clarity and conciseness, blots were cropped to the bands of interest. Full-length blots are depicted in Supplementary Fig. S12.

In order to gain a deeper insight into the mechanism behind the Nb-induced stabilisation of p53 in HeLa cells, we evaluated whether the p53-E6/E6AP complex was still efficiently formed. In SiHa cells, equal amounts of E6AP were co-precipitated by p53, irrespective of whether or not the p53 DBD Nbs were intracellularly expressed (Fig. 4a). This result is in agreement with the observations made in the CHX pulse chase assay (Fig. 3b). By contrast, substantially lower amounts of E6AP were co-precipitated by p53 in HeLa cells when p53 DBD Nbs were expressed (Fig. 4a). This observation was partially confirmed by the analysis of p53 ubiquitination, where a slight reduction in ubiquitinated forms of p53 was detected in the presence of the p53 DBD Nbs (Fig. 4b).

### A comparative analysis: p53 DBD Nbs vs SAHA

**Figure 5:**
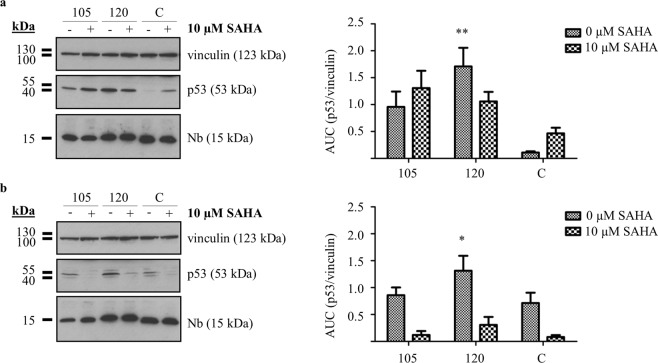
Evaluation of p53 levels in HPV-infected cells receiving a co-treatment with p53 DBD Nbs and the HDACi SAHA. HeLa (**a**) and SiHa **(b)** cells transfected with FLAG-tagged p53 DBD Nbs received (or did not receive) an additional treatment with 10 µM SAHA for a duration of 20 h. Cells transiently expressing a FLAG-tagged GFP Nb were implemented as negative control (C). Crude lysates (60 µg) were prepared and p53 levels were analysed through western blotting. Vinculin was used as a loading control and quantitative analysis of protein levels was accomplished by means of ImageJ. Values are represented as mean AUC (p53/vinculin) (±SEM) of 3 independent experiments. Statistical analysis was performed using a two-way ANOVA with a Bonferroni post-test. **(a)** p53 DBD Nbs were responsible for a stabilisation of p53 in HeLa cells, where significant higher p53 levels were observed for p53 DBD Nb120 compared to the negative control (p < 0.01). SAHA treatment of the control cells resulted in a 4.18-fold increase in p53 levels (C/− vs C/+). Nevertheless, the p53 DBD Nbs had a more substantial impact on p53 stability, irrespective of whether or not the cells were co-treated with SAHA. The stabilising effect of p53 DBD Nb105 was slightly enhanced in the presence of SAHA, represented by a 1.36-fold increase in p53 levels. By contrast, SAHA treatment of HeLa cells expressing p53 DBD Nb120 resulted in a 1.66-fold reduction of p53 levels. **(b)** Expression of p53 DBD Nb120 in SiHa cells caused a significant increase of p53 levels compared to the negative control (p < 0,05). Curiously, SAHA treatment had no impact on the stability of p53, neither in the control condition nor in SiHa cells expressing p53 DBD Nbs. Even more, treatment with SAHA rather promoted a reduction of p53 levels and counteracted the impact of the p53 DBD Nbs. SAHA treatment caused a 7.17-fold or a 4.23-fold reduction of p53 levels in cells expressing p53 DBD Nb105 or p53 DBD Nb120, respectively. Blots were cropped to the bands of interest. Full-length blots are depicted in Supplementary Fig. S13.

Suberoylanilide hydroximic acid (SAHA, also known as vorinostat) is a pan-histon deacetylase inhibitor (pan-HDACi) which received FDA-approval for the treatment of cutaneuous T-cell lymphoma^13^. The role of SAHA in the treatment of other cancers is being investigated and more recent discoveries support its use in HPV-related cancers. Interestingly, SAHA treatment of HPV-infected cells results in a stabilisation of p53 levels^14–16^. Since p53 DBD Nbs elicit a similar response, it was of interest to compare both approaches. For this purpose, transfected cells received (or did not receive) an additional treatment with 10 µM SAHA for a duration of 20 h. Thereafter, crude lysates were prepared and the impact of the compounds on p53 levels was determined by means of western blot (Fig. 5). The experiment was repeated 3 times. (for additional blots, please refer to Supplementary Fig. S6).

Treatment of HeLa cells with SAHA, resulted in a 4.18-fold augmentation of p53 levels as can be deduced by comparing p53 levels in the control condition in the presence or absence of SAHA treatment (Fig. 5a, compare lanes C/− with C/+). However, this effect did not match the impact observed after treatment of HeLa cells with p53 DBD Nbs alone. Quantitative analysis indicates that p53 reached a 2.09-fold (p53 DBD Nb105, 0 µM SAHA) and a 3.7-fold (p53 DBD Nb120, 0 µM SAHA) higher level when p53 DBD Nbs were expressed in HeLa cells compared to the SAHA-treated control cells (C, 10 µM SAHA) (Fig. 5a, compare lanes 105/− and 120/− with C/+). Additionally, we analysed whether co-treatment of HeLa cells with SAHA affected the impact of p53 DBD Nbs on p53 levels. In case of p53 DBD Nb105, co-treatment enhanced the Nb-induced effects, whereby we observed an additional increase of p53 levels with a factor of 1.36. By contrast, co-treatment negatively impacted the effect induced by p53 DBD Nb120, reflected by a 1.66-fold decrease in p53 levels (Fig. 5a, compare lanes 105/− with 105/+ and 120/− with 120/+).

Curiously, SAHA-treatment of SiHa cells seemed to have a negative impact on p53 levels. In the control cells, SAHA-treatment resulted in an 8.88-fold reduction of p53 levels, rather than causing a stabilisation of p53. Moreover, SAHA appeared to antagonise the impact of the p53 DBD Nbs on p53 levels. In case of p53 DBD Nb105, co-treatment with SAHA led to a 7.17-fold reduction of p53 levels. This phenomenon was also observed for p53 DBD Nb120, where co-treatment with SAHA caused a 4.23-fold reduction of p53 levels (Fig. 5b, compare lanes 105/− with 105+ and 120/− with 120/+).

### Assessment of the functional impact of p53 stabilisation in HeLa cells

Experiments reported here clearly demonstrate that p53 DBD Nbs stabilise endogenous p53 in HPV-infected HeLa cells. We next addressed the question whether this stabilisation has functional consequences. First, it was assessed whether or not p53 is enriched in the nucleus and exerts its function as a transcriptional activator. This was examined through a combination of immunocytochemistry and transactivation assays. Second, it was also determined whether stabilisation of p53 impacts cell viability. This was analysed by means of a XTT assay.

#### Cellular distribution of p53

**Figure 6:**
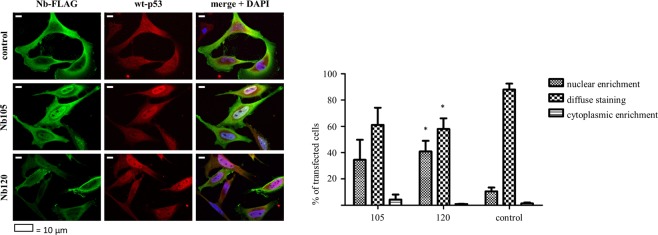
Endogenous p53 accumulates in the nucleus of HeLa cells after stabilisation by p53 DBD Nb120. Representative epifluorescent images displaying the cellular distribution pattern of endogenous p53 in Nb-transfected HeLa cells. HeLa cells transiently expressed a FLAG-tagged p53 DBD Nb or a FLAG-tagged GFP Nb, representing the negative control (control). Immunostaining was performed 24 h after transfection. The images demonstrate changes in the cellular distribution pattern of endogenous p53 after Nb-mediated stabilisation of the tumor suppressor. A largely diffuse staining pattern of p53 was detected in the control condition, where nuclear enrichment of p53 was only occasionally observed. By contrast, p53 displays a different cellular distribution pattern after its stabilisation by the p53 DBD Nbs. Although a diffuse staining pattern is also frequently observed, a substantial larger fraction of the transfected cells display nuclear enriched p53. In the presence of p53 DBD Nb120, nuclear enrichment of p53 was detected in a significantly higher percentage of the transfected cells (p < 0,05). FLAG-tagged Nbs and endogenous p53 were visualised with an anti-FLAG antibody and a DO-1 antibody, respectively. Visualisation of the nucleus was achieved with DAPI. Cellular distribution of p53 was analysed by means of ImageJ. Values represent the mean % of transfected cells (±SEM) of 3 independent experiments. A total of ≥100 transfected cells were analysed for each independent experiment. Statistical analysis was performed via a two-way ANOVA with Bonferroni post-test.

The tumour suppressor p53 is well-known for its role in preventing cellular transformation. This is largely established through its functionality as a transcriptional transactivator, which allows p53 to regulate the expression of a multitude of genes, thereby initiating an appropriate response to cellular stress signals. In order to pursue its function as a transcriptional transactivator, the protein needs to translocate to the nucleus. To gain insight into the cellular distribution pattern of p53 in HeLa cells, an immunostaining was performed. In the control condition (cells transfected with a GFP Nb) p53 staining was weak, which corresponds to its rather low expression levels. Nevertheless, the protein was distributed evenly throughout the cell. By contrast, when p53 was stabilised by the action of a p53 DBD Nb, it frequently displayed a strong nuclear staining pattern. In the presence of p53 DBD Nb120, a significantly higher percentage of the transfected cells displayed nuclear enrichment of p53 compared to the control cells. Nuclear enriched p53 was detected in 41% of the transfected cells (p < 0,05). The tumor suppressor thus preferentially accumulates in the nucleus after Nb-mediated stabilisation (Fig. 6). Data originate from 3 independent experiments, whereby the cellular distribution of p53 in each condition was analysed for at least 100 transfected cells.

#### Perturbation of the transactivation functions of p53

**Figure 7:**
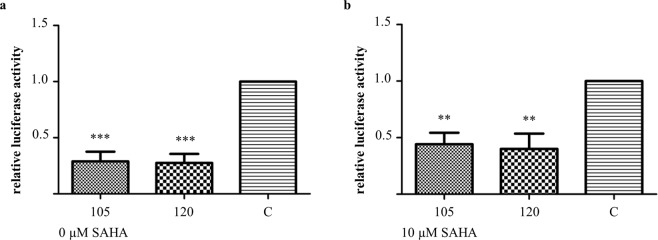
p53 DBD Nbs have an inhibitory effect on the transcriptional activity of p53. The functionality of p53 as a transcriptional transactivator was evaluated by means of a transactivation assay. HeLa cells were co-transfected with a FLAG-tagged p53 DBD Nb and a pGL13 luciferase reporter. The luciferase activity is representative for the activity of p53 as transcriptional transactivator. A negative control (C) was included whereby HeLa cells were co-transfected with a FLAG-tagged GFP Nb and the pGL13 luciferase reporter. The transfected cells received (or did not receive) an additional treatment with 10 µM SAHA for a duration of 20 h. Luciferase activity was measured 48 h after transfection. Values represent the mean luciferase activity relative to the negative control (±SEM) measured for triplicate samples in 4 independent experiments. Statistical analysis was performed via a one-way ANOVA, with Dunnett’s multiple comparison test with a p-value < 0.05. **(a)** In the absence of SAHA treatment, the p53 DBD Nbs were responsible for a significant reduction of the transactivation activity of p53 compared to the control condition. The relative luciferase activity was reduced by 71% and 72% after transient expression of p53 DBD Nb105 and p53 DBD Nb120, respectively (p < 0.001) **(b)** Similar observations were made when the transfected HeLa cells received the co-treatment with 10 µM SAHA. In the presence of p53 DBD Nb105, the relative luciferase activity was reduced by 56%. When p53 DBD Nb120 was expressed, the luciferase activity was lowered with 60% (p < 0.01).

Next, the functionality of p53 as a transcriptional transactivator was evaluated by means of transactivation assays. HeLa cells were co-transfected with a p53 DBD Nb and a pGL13 luciferase construct which contains a luciferase reporter gene that is preceded by 13 repeats of the p53 consensus sequence. A negative control was implemented whereby cells were co-transfected with a GFP Nb and the pGL13 luciferase construct. Transfected cells received (or did not receive) an additional treatment with 10 µM SAHA. Interestingly, the transactivation functions of p53 were disrupted in the presence of a p53 DBD Nb, irrespective of whether the cells received (or did not receive) the additional treatment with SAHA. In comparison with the negative control, the relative luciferase activity dropped significantly in the presence of p53 DBD Nbs, whereby p53 DBD Nb105 and p53 DBD Nb120 were responsible for a reduction of 71% and 72%, respectively (p < 0.001). The observed effect persisted when the transfected cells received the co-treatment with 10 µM SAHA. Here, the luciferase activity measured for p53 DBD Nb105 was 56% lower compared to the negative control (p < 0.01). In case of p53 DBD Nb120, a reduction of 60% was observed (p < 0.01) (Fig. 7). Data originate from 4 independent experiments.

The transactivation assay was repeated using U2OS pGL13 cells which express the pG13 luciferase construct in a stable manner. The objective was to verify whether the observed perturbation of the transactivation functions of p53 is specific for HPV-infected cells or whether it concerns a more general phenomenon. Cells were transfected with a p53 DBD Nb or a GFP Nb. An additional positive control was implemented, whereby cells were transfected with a construct that encodes HA-tagged R175H p53 mutant. This hotspot mutant exerts a dominant-negative effect over wild type p53^17^. The transfected cells received (or did not receive) an additional treatment with 5 µM Nutlin3a, which is a Mdm2 antagonist known to activate p53. As was the case in HeLa cells, the p53 DBD Nbs exerted an inhibitory effect over the transactivation functions of p53, whereby the relative luciferase activity was reduced to the same extent as seen for HA-tagged R175H p53 mutant. By comparison with the negative control, the relative luciferase activity was reduced with 43% (p53 DBD Nb105, p < 0.001), 33% (p53 DBD Nb120, p < 0.01) and 35% (R175H p53 mutant, p < 0.01). When p53 was activated by means of treatment with Nutlin3a, the inhibitory effect of the constructs remained. More specifically, a reduction of 48% (p53 DBD Nb105, p < 0.001), 49% (p53 DBD Nb120, p < 0.001) and 51% (R175H p53 mutant, p < 0.001) was observed. Data originate from 4 independent experiments (Supplementary Fig. S7).

The transfection efficiency of the constructs was evaluated for each individual repeat, both for HeLa cells and U2OS pGL13 cells. The corresponding western blot data are depicted in supplementary materials (Supplementary Fig. S8).

#### p53 DBD Nbs elicit increased cell proliferation and viability of HeLa cells

**Figure 8:**
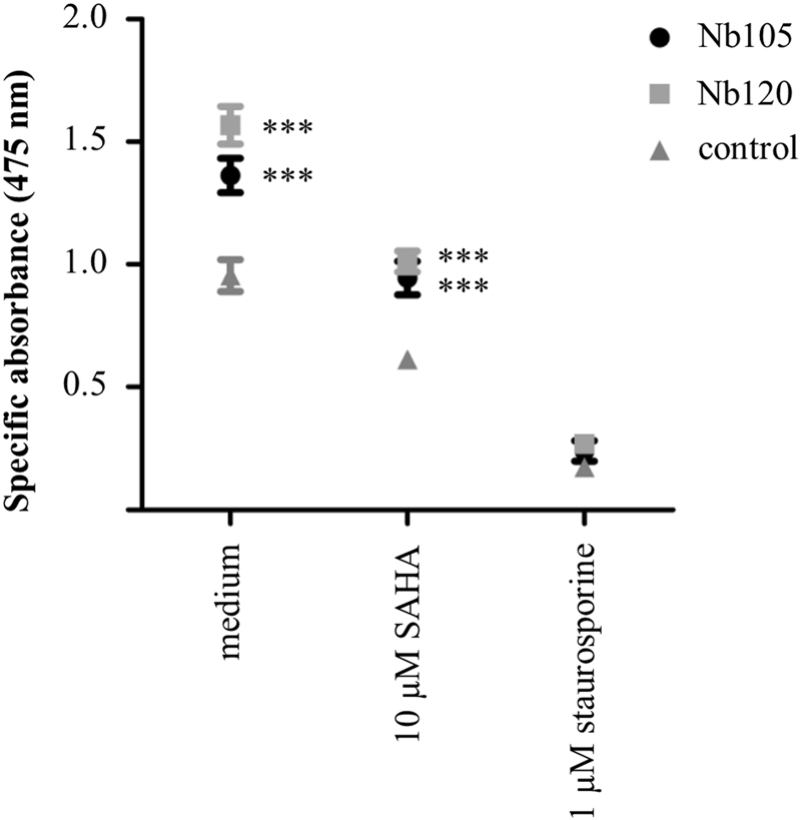
HeLa cells display an increased cellular proliferation and viability when p53 DBD Nbs are expressed. An XTT assay was performed to evaluate whether or not the p53 DBD Nb-induced stabilisation of p53 levels affects the cell viability of HeLa cells. The cells were transfected with FLAG-tagged p53 DBD Nbs or a FLAG-tagged GFP Nb (control). Thereafter, the transfected cells received (or did not receive) an additional treatment with 10 µM SAHA or 1 µM staurosporine for a duration of 20 h. The graph depicts the mean net absorbance of the formazan dye measured at 475 nm (±SEM) of triplicate samples in 4 independent experiments. Statistical analysis was performed by means of a two-way ANOVA with a Bonferroni post-test. A significant higher absorbance was measured for HeLa cells expressing the p53 DBD Nbs (p < 0.001). This result implicates that expression of p53 DBD Nbs coincided with an increased proliferation and viability of HeLa cells. When the cells were co-treated with 10 µM SAHA, there was a decline in cellular proliferation. However, there was still a significant difference between HeLa cells expressing a p53 DBD Nb and the control condition (p < 0.001). The cell viability was dramatically reduced for all conditions when the cells were co-treated with 1 µM staurosporine.

A 2,3-bis-(2-methoxy-4-nitro-5-sulfophenyl)-2H-tetrazolium-5-carboxyanilide (XTT) assay was performed to evaluate whether the Nb-induced increase of p53 levels has an impact on cell viability. Notwithstanding the fact that the functionality of p53 as a transcriptional transactivator is perturbed in the presence of p53 DBD Nbs, cell viability can still be affected as a result of the transcription-independent functions of p53^18^. Transfected cells were plated in triplicate in 96-well microtiter plates. Thereafter, cells received (or did not receive) an additional treatment with 10 µM SAHA or 1 µM staurosporine. The latter compound served as a positive control, since the broad spectrum kinase inhibitor is a known inducer of apoptosis^19^. The experiment was repeated 4 times. Remarkably, in the presence of p53 DBD Nbs, HeLa cells demonstrated a significant higher cell proliferation compared to the control cells (p53 DBD Nb105 and p53 DBD Nb120: p < 0.001). This was still the case when cells received the co-treatment with 10 µM SAHA (p53 DBD Nb105: p < 0.01, p53 DBD Nb120: p < 0.001). However, a high degree of apoptosis was observed for all conditions when the cells were treated with 1 µM staurosporine (Fig. 8). The transfection efficiency was evaluated for each individual repeat (Supplementary Fig. S9).

## Discussion

HPVs are non-enveloped viruses with a double stranded circular genome of ±8 kB that infect mucosal and cutaneous epithelia. Based on DNA sequence analysis, HPVs can be subdivided into 5 genera which display different life-cycle characteristics and disease associations. Within the alpha genera, the mucosal types are classified into low-risk HPVs and high-risk HPVs. Persistent infection with high-risk HPVs is associated with tumour development. There are 12 characterized high-risk HPVs, of which HPV16 and HPV18 are responsible for 71% of all cervical cancers^3,20^. High-risk HPV-induced tumorigenesis is an unwanted side effect resulting from the variety of replication and immune evasion strategies that viruses have developed to establish long-term infections. Amplification of the viral genome and synthesis of the viral progeny occurs in the terminally differentiated layers of the epithelium. Under normal circumstances, these cells do not express DNA replication enzymes but this is circumvented via viral targeting of several cellular regulatory pathways, like the pRb- and p53 pathway amongst others^21^. Deregulation of these pathways is achieved by the actions of the viral E6 and E7 oncoproteins. Other cellular targets of these oncoproteins are nicely reviewed elsewhere^7,8,22^. The viral E6 protein targets p53 for excessive proteasomal degradation which renders the tumour suppressor unable to induce apoptosis in response to the abnormal cellular proliferation caused by E7-mediated inhibition of pRb. Interestingly, despite the fact that both pathways are kept dormant, their functionality appears to be intact^23^. By consequence, E6 and E7 thus constitute interesting targets for the development of new targeted drugs. This rationale has been exploited by several researchers who have investigated the efficacy of targeting E6 and E7 by means of immunotherapeutic approaches^24,25^, CRISPR/Cas9-based strategies^26^, siRNA^27–30^, and peptides, intrabodies or small molecule inhibitors^31–36^. In this study, an alternative approach is explored. We developed Nbs against the DBD of p53 in order to evaluate whether they are capable of enhancing the stability of the tumour suppressor in HPV-infected cells. The intracellular stability and functionality of the p53 DBD Nbs was confirmed by means of an *in vivo* pull down assay (Fig. 1). Not all antibody formats can be used to target intracellular proteins, since the reducing intracellular environment does not allow the formation of inter- and intramolecular disulphide bridges^37^. Nbs however display a high stability and solubility compared to conventional antibody fragments^12^. Interestingly, intracellular expression of the p53 DBD Nbs in HeLa cells (HPV18) and SiHa cells (HPV16) resulted in increased p53 levels. This effect appeared to be specific for HPV-infected cells, since a comparable observation could not be made for HPV-negative U2OS cells (Fig. 2). Furthermore, p53 DBD Nb105 and p53 DBD Nb120 substantially prolonged p53 half-life in HeLa cells, which indicates that the Nbs are responsible for p53 stabilisation (Fig. 3 and Supplementary Fig. S4). Although Mdm2-induced degradation of p53 appears to be inactive in HPV-infected cells, several other E3 ubiquitin ligases exist which are known to be involved in p53 degradation^38,39^. However, the observed stabilisation of p53 in HPV-infected cells is most likely the result of a perturbing effect of the p53 DBD Nbs on the E6/E6AP-induced proteasomal degradation of p53, since other E3 ubiquitin ligases involved in p53 degradation are universally expressed. Indeed, substantially lower amounts of E6AP are co-precipitated by p53 in HeLa cells when the p53 DBD Nbs were intracellularly expressed (Fig. 4a). To obtain an idea about the efficiency by which the p53 DBD Nbs induce p53 stabilisation, a comparison was made with the HDACi SAHA, also known as Vorinostat (Fig. 5). Treatment of cervical cancer cells with SAHA results in an augmentation of p53 levels, although the underlying mechanism is not entirely clear. It has been suggested that SAHA treatment inhibits deacetylation of C-terminal lysine residues, thereby rendering them inaccessible for polyubiquitination^14^. In addition, there is also evidence that SAHA treatment decreases E6 and E7 protein levels by inhibiting the transcription initiation of the viral genes^16^. SAHA treatment of HeLa cells indeed resulted in a stabilisation of p53 levels, where a 4.18-fold increase in p53 levels was observed. Nevertheless, the p53 DBD Nbs appear to be stabilise p53 more efficiently, since p53 levels are 2.09-fold (p53 DBD Nb105) and 3.7-fold (p53 DBD Nb120) higher than observed for SAHA-treated control cells. It was also investigated whether co-treatment of HeLa cells would result in an additional augmentation of p53 levels. This was the case for p53 DBD Nb105, but resulted in a slight decrease in p53 levels for HeLa cells expressing p53 DBD Nb120. Nonetheless, under any circumstances p53 levels were higher than observed for SAHA-treated control cells (Fig. 5a). The results obtained for SiHa cells were less straightforward, since SAHA treatment reduced p53 levels. Even more, despite the fact that the expression levels of the p53 DBD Nbs were unaffected, p53 levels declined when cells were co-treated with SAHA (Fig. 5b). Apparently, HDACi can also suppress p53 transcription via the inhibition of HDAC8. This reduces HoxA5 expression and thereby interferes with HoxA5-induced transcription of p53^40^. A differential expression pattern of HDAC8 or HoxA5 might explain the differential effects of SAHA treatment between HeLa cells and SiHa cells. Nevertheless, this observation stresses the importance of the cellular context in which HDACi are used. Co-treatment of SiHa cells with p53-activating agents and a HDACi would be counterproductive, whilst HeLa cells might benefit from such strategy.

Next, it was verified whether the stabilised p53 is active and thus capable of inducing an appropriate response to unchecked cell proliferation. It has been demonstrated that HPV-infected cells can respond to genotoxic stresses, whereby apoptotic cell death is induced by means of p53-dependent and –independent pathways^41^. Proof of concept is also found in studies demonstrating that targeting E6 by means of siRNA, small compounds or intrabodies results in a reduced cellular proliferation and the induction of apoptosis^30,32,35^. First, the cellular distribution pattern of endogenous p53 was investigated via immunocytochemistry, since E6 can sequester p53 in the cytoplasm by masking its C-terminal nuclear localization signal (NLS) or by inducing a hyperactive nuclear export^42,43^. In HeLa cells, the stabilisation of p53 by the action of p53 DBD Nbs is associated with a shift in the cellular distribution pattern of the tumour suppressor. Indeed, nuclear enrichment of p53 was observed for a substantial fraction of the transfected cells (Fig. 6). Second, a transactivation assay demonstrated that the p53 DBD Nbs inhibit the transcriptional program of p53 (Fig. 7 and Supplementary Fig. S7). Moreover, in U2OS pGL13 cells, the p53 DBD Nbs inhibit the transcriptional activity of p53 as efficiently as the R175H p53 mutant which exerts a dominant negative effect over wild type p53-mediated transcription^17^. We previously discovered a p53 DBD Nb capable of inhibiting the transcriptional activity of p53^44^. The Nb bound p53 on the opposite side of the DNA-binding surface, leaving the functional architecture of the tumour suppressor intact. Interestingly, the E6 protein also targets p53 at a different site than the DNA-binding surface^9^. We therefore, do not exclude the possibility that the mere binding of E6 with p53 could be sufficient to render the tumour suppressor inactive, similar to the Nb. This might be an additional mechanism whereby E6 mediates the functional inactivation of p53, apart from its inhibitory effect on p300/CBP, a transcriptional co-activator of p53^45^. Alternatively, it is also possible that the Nbs lock the tumour suppressor in a conformation that is similar to what is observed for several p53 mutants, which also lack transcriptional activity^46,47^.

Finally, it was also investigated whether the increased p53 levels have an impact on cell viability of HeLa cells by means of an XTT-assay. Aside from its functionality as a transcriptional transactivator, p53 also exerts transcription-independent functions in the cytoplasm which could impact cell viability^18^. HeLa cells expressing p53 DBD Nbs exhibited increased cellular proliferation compared to control cells (Fig. 8). There are thus no indications that p53 is capable of exerting its transcription-independent functions in the cytoplasm, which would for instance result in the induction of apoptosis or necrosis^48,49^. This is not surprising, given the intertwinement of the nuclear and cytoplasmic functions of p53, whereby several p53-regulated proteins contribute to the cytoplasmic functions of p53^50^. The negative impact of the p53 DBD Nbs on the functionality of p53 as a transcriptional transactivator, thus might be a double-hit. By contrast, cell viability is substantially reduced when HeLa cells are additionally treated with SAHA or staurosporine and this effect appears to be independent from Nb expression (Fig. 8). Both compounds are potent inducers of apoptosis, whereby SAHA promotes Bim-mediated apoptotic pathways in HPV-infected cells in a p53-independent way and staurosporine induces apoptosis via caspase-dependent and –independent pathways^14,51^. Given the strong impact of the p53 DBD Nbs on the functionality of p53, it would be interesting to identify the exact epitopes targeted by the Nbs and to unravel the mechanism behind p53 inactivation. A detailed structural insight of the targeted epitopes could be obtained via X-ray crystallography or via faster alternative screening techniques like hydrogen/deuterium exchange MS or NMR spectroscopy^52,53^. Unravelling the mechanisms behind p53 inactivation can however be a daunting task, since p53 forms the central hub of an intricate molecular network and is thus involved in numerous protein-protein interactions. However, a combination of Nb footprinting and Bio-ID proximity labelling, can be an elegant strategy to identify (missing) interaction partners. Interaction between a Nb and its target might result in the displacement of specific interaction partners. Each Nb will have its own specific footprint, thus displaced interaction partners can be identified by comparing specific footprints generated by Nbs targeting a different epitope on the same antigen^54,55^. Combination of this strategy with the Bio-ID proximity labelling would create a very powerful tool through which even weak and transient interactions could be identified. Interestingly, it has been recently demonstrated that this labelling strategy can be used for proximal protein mapping for endogenous p53^56^.

In summary, p53 DBD Nbs are responsible for a substantial stabilisation of p53 levels in HPV-infected HeLa cells. This stabilisation might be the result of a perturbation of the viral E6-mediated proteasomal degradation of p53. Nevertheless, despite being more strongly nuclear enriched, p53 is not capable of exerting its function as a transcriptional transactivator due to an inhibitory effect exerted by the p53 DBD Nbs. Even more, through the inactivation of p53’s transcriptional activity, p53 DBD Nb’ expressing HeLa cells acquire a proliferative advantage. Therefore, we believe it is of interest to identify the epitopes targeted by the p53 DBD Nbs and to gain insight in the mechanisms by which p53 inactivation is realised.

## Materials and Methods

### Antibodies and reagents

See Supplementary Methods for details on the used antibodies and reagents.

### Generation of p53 DBD Nbs

See Supplementary Methods for details on the generation of p53 DBD Nbs.

All animal work was performed by the VIB Nanobody Service Facility in accordance with relevant guidelines and regulations and has Public Health Service-approved animal welfare assurance from the Office of Laboratory Animal Welfare (F16-00131 (A5593-01)).

### cDNA cloning

See Supplementary Methods for a detailed description of the cloning reaction.

### Cell culture and transfection

HeLa, SiHa, HEK293T, U2OS and U2OS pGL13 cell lines were cultivated in DMEM (Gibco, Thermo Fisher Scientific) supplemented with 10% fetal bovine serum. The cells were grown at 37 °C in a humidified incubator at 5% CO_2_ (HeLa and SiHa cells) or 10% CO_2_ (HEK293T, U2OS and U2OS pGL13 cells). Cell cultures were frequently tested and found negative for mycoplasma contamination using the HEK-Blue detection kit from Invivogen. The JetPrime transfection reagent (Polyplus Transfection) was used to transiently express the Nbs in the aforementioned cell lines. Transfection was performed according to the manufacturers’ instructions.

### *In vivo* pull-down assay

The *in vivo* pull-down assay was performed as previously described^57^. In short, p53 DBD Nbs were transiently expressed in HEK293T cells. A negative control was implemented whereby cells transiently expressed an unrelated Nb, GFP Nb, targeted against the green fluorescent protein (GFP). Crude lysates were prepared using an ice-cold Tris-lysis buffer (20 mM Tris-HCl, 150 mM NaCl, 1% Triton X-100, 1 mM phenyl-methane sulfonyl fluoride (PMSF) and 200 µg/ml protease inhibitor cocktail mix). Next, 1 mg of crude lysate was incubated with 10 µl settled anti-FLAG M2 affinity gel. The samples were rotated for 1 h at 4 °C, resulting in the immobilisation of the FLAG-tagged Nbs onto the affinity gel. After thorough washing, performed with the Tris-lysis buffer, the bound proteins were eluted by boiling the samples for 5 min in Laemmli sample buffer. Proteins were separated according to their molecular weight via SDS-page, which was followed by western blot analysis. Expression levels of the transfected FLAG-tagged Nbs were evaluated by analysing a 40 µg sample of the crude lysate via SDS-page and western blotting. For the visualisation of the FLAG-tagged Nbs and p53, rabbit polyclonal anti-FLAG (F7425) and mouse monoclonal anti-p53 (DO1, P6874) were employed, respectively. Beta-actin was implemented as loading control and was visualised via goat polyclonal anti-beta actin (ab8229).

### Evaluating the influence of p53 DBD Nbs on the stability of p53 in HPV-infected cells

#### Assessment of p53 levels

p53 DBD Nbs were transiently expressed in HeLa cells and SiHa cells. A negative control was implemented whereby cells were transfected with a GFP Nb. Cell lysis was performed 24 h after transfection. Thereafter, a 60 µg sample of crude lysate was loaded onto a 15% SDS-gel and proteins were separated according to their molecular weight, followed by western blot analysis. For the visualisation of the FLAG-tagged Nbs and p53, rabbit polyclonal anti-FLAG (F7425) and mouse monoclonal anti-p53 (DO1, P6874) were employed, respectively. Vinculin was implemented as loading control and was visualised via mouse monoclonal anti-vinculin (clone hVIN-1, V9131). Western blot data were quantified using ImageJ (National Instutites of Health, Bethesda, MD, USA). In order to evaluate whether the observed effects were specific for HPV-infected cells, the experiment was repeated for non HPV-infected U2OS cells.

Similar strategy was employed when investigating the influence of SAHA co-treatment on p53 DBD Nb-induced variations in p53 levels for HeLa cells and SiHa cells. The cells received (or did not receive) the additional treatment with 10 µM SAHA 4 h after transfection.

#### Cycloheximide pulse chase assay

p53 DBD Nbs or a GFP Nb, implemented as a negative control, were transiently expressed in HeLa cells and SiHa cells. Novel protein synthesis was inhibited 24 h after transfection by treating the cells with 20 µg/ml cycloheximide (CHX). In order to evaluate the half-life of p53, cell lysis was performed at different time-points after CHX treatment (i.e. 0 h, 0.5 h, 1 h, 2 h, 4 h and 6 h). Thereafter, a 60 µg sample of crude lysate was loaded onto a 15% SDS-gel for each indicated time-point and proteins were separated according to their molecular weight and analysed through western blot. For the visualisation of the FLAG-tagged Nbs and p53, rabbit polyclonal anti-FLAG (F7425) and mouse monoclonal anti-p53 (DO1, P6874) were employed, respectively. Vinculin was implemented as loading control and was visualised via mouse monoclonal anti-vinculin (clone hVIN-1, V9131). Western blot data were quantified using ImageJ (National Instutites of Health, Bethesda, MD, USA).

#### Co-immunoprecipitation assay: evaluation of the p53-E6/E6AP interaction and p53 ubiquitination

HeLa and SiHa cells were transfected with a FLAG-tagged p53 DBD Nb or the FLAG-tagged GFP Nb and were treated 24 h later with 10 µM (HeLa) or 25 µM (SiHa) MG-132 for a duration of 2 h. Cell lysis was performed using an ice-cold Tris-lysis buffer (20 mM Tris-HCl, 150 mM NaCl; 1% Triton X-100, 1 mM PMSF, 200 µg/ml protease inhibitor cocktail mix, 5 mM EDTA and 1 mM EGTA). When assessing p53 ubiquitination, 10 mM N-ethylmaleimide was additionally included in the lysisbuffer. Next, p53 was immunoprecipitated, using DO-1 (5 µg antibody/1 mg of crude lysate) and protein G sepharose beads. To exclude non-specific binding of p53 with the beads, an additional negative control was implemented, where 1 mg of crude lysate of MG132-treated non-transfected cells was incubated with protein G sepharose beads in the absence of DO-1. After incubation, beads were washed with Tris-lysis buffer and boiled for 5 min in Laemmli SDS sample buffer to elute the proteins. The proteins were separated according to their molecular weight via SDS-PAGE (8% SDS-gel), which was followed by western blot analysis. The expression levels of the transfected constructs were evaluated in 60 µg of the crude lysates (15% SDS-gel). E6AP was visualised by means of rabbit monoclonal anti-UBE3A (ab126765). For the visualisation of the FLAG-tagged Nbs and p53, rabbit polyclonal anti-FLAG (F7425) and mouse monoclonal anti-p53 (DO1, P6874) were employed, respectively. Vinculin was implemented as loading control and was visualised via mouse monoclonal anti-vinculin (clone hVIN-1, V9131).

### Assessment of the functional impact of p53 stabilization

#### Immunofluorescence and microscopy

Immunostaining was performed as earlier described^58^. In a nutshell, HeLa cells were seeded onto collagen-coated coverslips and were transfected 24 h after cell-seeding with FLAG-tagged p53 DBD Nbs or a FLAG-tagged GFP Nb. The immunostaining was performed another 24 h later. Polyclonal rabbit anti-FLAG (F7425, Sigma) and monoclonal mouse anti-p53 (DO-1) (P6874, Sigma) were used for the visualisation of the FLAG-tagged Nbs and p53, respectively. Staining of the nucleus was performed using 4′,6-diamidino-2-phenylindole (DAPI) (D8417, Sigma). Coverslips were mounted on microscopic slides using 1% n-propyl gallate in glycerol as mounting medium and were sealed with nail polish. Analysis of the cells was performed with a Zeiss Axiovert 200 M fluorescence microscope with Apotome module (Zeiss x63 1.4-NA Oil Plan-Apochromat objective, Carl Zeiss, Oberkochen, Germany) and Axiovision 4.5 software (Zeiss). Cellular distribution of p53 was examined via ImageJ (National Instutites of Health, Bethesda, MD, USA).

#### Transactivation assay

FLAG-tagged p53 DBD Nbs were co-expressed with a pGL13 luciferase construct in HeLa cells, which were seeded into a T25 culture flask. A negative control was implemented, where cells transiently express a FLAG-tagged GFP Nb. Cells were re-seeded 24 h after transfection into a 96-well format (15.000 cells/well). Transfected cells received (or did not receive) an additional treatment with 10 µM SAHA for a duration of 20 h. In order to evaluate whether the effects are specific for HPV-infected cells, the experiment was also performed using U2OS pGL13 cells that express the pGL13 luciferase construct in a stable manner. Here, an additional positive control was implemented, which consists of cells transiently expressing HA-tagged mutant p53 (R175H) (pcDNA3.1 vector). Transfected U2OS pGL13 cells received (or did not receive) an additional treatment with 5 µM Nutlin3a for a duration of 20 h. Triplicate samples were provided for each condition. Luciferase activity was measured the next day as previously described^57^. Measurements were executed using a Topcount luminometer (Canberra-Packard). A total of 4 independent experiments were performed. The transfection efficiency of the transfected constructs was evaluated for each individual experiment. Therefore, crude lysates were prepared from the remainder of the cells (collected after re-seeding the cells into a 96-well format) and 60 µg (HeLa cells) or 40 µg (U2OS pGL13 cells) samples were subsequently analysed via SDS-page and western blotting. For the visualisation of the FLAG-tagged Nbs, rabbit polyclonal anti-FLAG (F7425) was used. Endogenous p53 was visualised using mouse monoclonal anti-p53 (DO1, P6874), whilst HA-tagged mutant p53 (R175H) was detected via mouse monoclonal anti-HA (clone 12CA5, 11583816001). Vinculin was implemented as loading control and was visualized via mouse monoclonal anti-vinculin (clone hVIN-1, V9131).

#### XTT assay

The XTT assay was performed as previously described and was executed according the manufacturer’s instructions^57^. HeLa cells were re-seeded 24 h after transfection with FLAG-tagged p53 DBD Nbs or a FLAG-tagged GFP Nb into a 96-well format, where 100 µl of the cell suspension (at a dilution of 2.5 * 10^5^ cells/ml) was added per well (triplicate samples). Blank background control wells, containing only cell medium, were also provided. Next, the cells received (or did not receive) an additional treatment with 10 µM SAHA or 1 µM staurosporine for a duration of 20 h. Thereafter, 50 µl of activated XTT-solution was added to the wells and the absorbance of the samples was measured 4 h later at 475 nm (specific readings) and 660 nm (non-specific readings). The specific absorbance is obtained after subtracting the average absorbance measurements for the blank background control wells and the average values for the non-specific readings from the specific readings measured at 475 nm. The effect of each construct was studied in 4 independent experiments. In order to evaluate the transfection efficiency, 60 µg samples of crude lysate were prepared from the remainder of the cells (collected after re-seeding the cells into a 96-well format) which were subsequently analysed via SDS-page and western blotting. For the visualisation of the FLAG-tagged Nbs and p53, rabbit polyclonal anti-FLAG (F7425) and mouse monoclonal anti-p53 (DO1, P6874) were employed as primary antibodies, respectively. Vinculin was implemented as loading control and was visualised via mouse monoclonal anti-vinculin (clone hVIN-1, V9131).

## Supplementary information

Supplementary Information

## Data Availability

All data generated or analysed during this study are included in this published article (and its Supplementary Information Files). p53 cDNA nanobody sequences are licensed to Gulliver Biomed BVBA.
