# Supplementary material for: Nb-induced stabilisation of p53 in HPV-infected cells
Source: Sci Rep. 2019 Sep 3;9:12680. doi: 10.1038/s41598-019-49061-9 (PMC6722090; doi:10.1038/s41598-019-49061-9)
Supplement: Supplementary file 1 — Supplementary Information [file 41598_2019_49061_MOESM1_ESM.pdf]

Nb-induced stabilisation of p53 in HPV-infected cells

Authors
Anneleen Steels[1], Laura Vannevel[1], Olivier Zwaenepoel[1] and Jan Gettemans*[1]

Affiliations
[1]Department of Biomolecular Medicine, Faculty of Medicine and Health Sciences, Campus Rommelaere, A. Baertsoenkaai 3, Ghent University, Ghent, Belgium.

*Corresponding author:

Department of Biomolecular Medicine,
Faculty of Medicine and Health Sciences,
Campus Rommelaere, Ghent University
Albert Baertsoenkaai 3
B-9000 Ghent, Belgium

Tel: + 32 9 2649340
Fax: + 32 9 2649490
E-mail: jan.gettemans@ugent.be

Supplementary Figures

Supplementary Figure S1

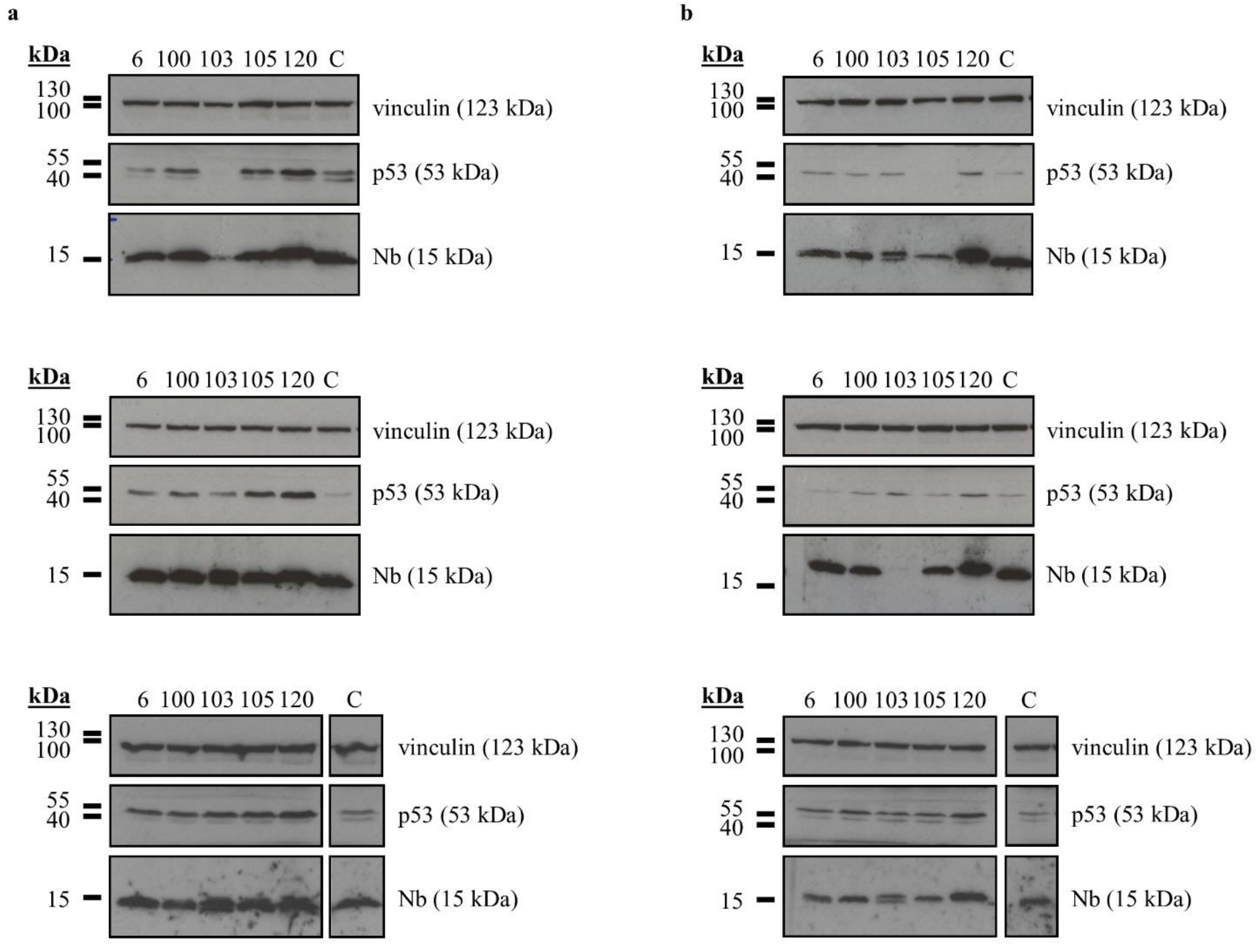

Supplementary Figure S1: significant increase of endogenous p53 levels in HPV-infected cells in the presence of p53 DBD Nbs. The influence of transiently expressed p53 DBD Nbs on p53 expression levels was examined for HeLa **(a)** and SiHa **(b)** cells. Cells were transfected with FLAG-tagged p53 DBD Nbs or a FLAG-tagged GFP Nb, which was implemented as a negative control (C). Crude lysates (60 µg) were prepared 24h post-transfection and protein levels were analysed via western blot. The western blot data of 3 additional executed repetitions of the experiment are depicted here. Data originating from 4 independent repetitions were used to quantitate nanobody-induced alterations of p53 expression levels. Vinculin was used as a loading control. For reasons of clarity and conciseness, blots were cropped to the bands of interest.

Supplementary Figure S2

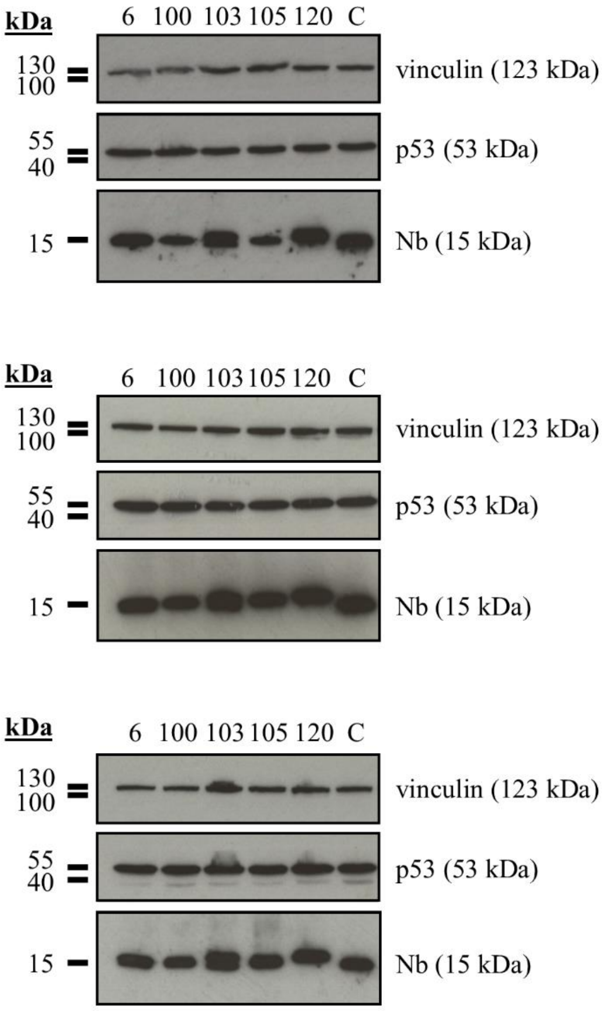

Supplementary Figure S2: endogenous p53 levels in U2OS cells are unaffected by the presence of p53 DBD Nbs. In order to evaluate whether there is an association between the Nb-induced alterations of p53 levels in HeLa and SiHa cells and the fact that both cell lines are infected by HPV, the experimental set-up was applied to U2OS cells (HPV-negative). U2OS cells transiently expressed a FLAG-tagged p53 DBD Nb or an unrelated FLAG-tagged GFP Nb which served as negative control (C). Crude lysates (40 µg) were prepared 24h post-transfection and protein expression levels were evaluated via western blot. The western blot data of 3 additional executed experiments are depicted here. Data originating from 4 independent repetitions were used to quantitate Nb-induced alterations of p53 expression levels. Vinculin was used as a loading control. For reasons of clarity and conciseness, blots were cropped to the bands of interest.

Supplementary Figure S3

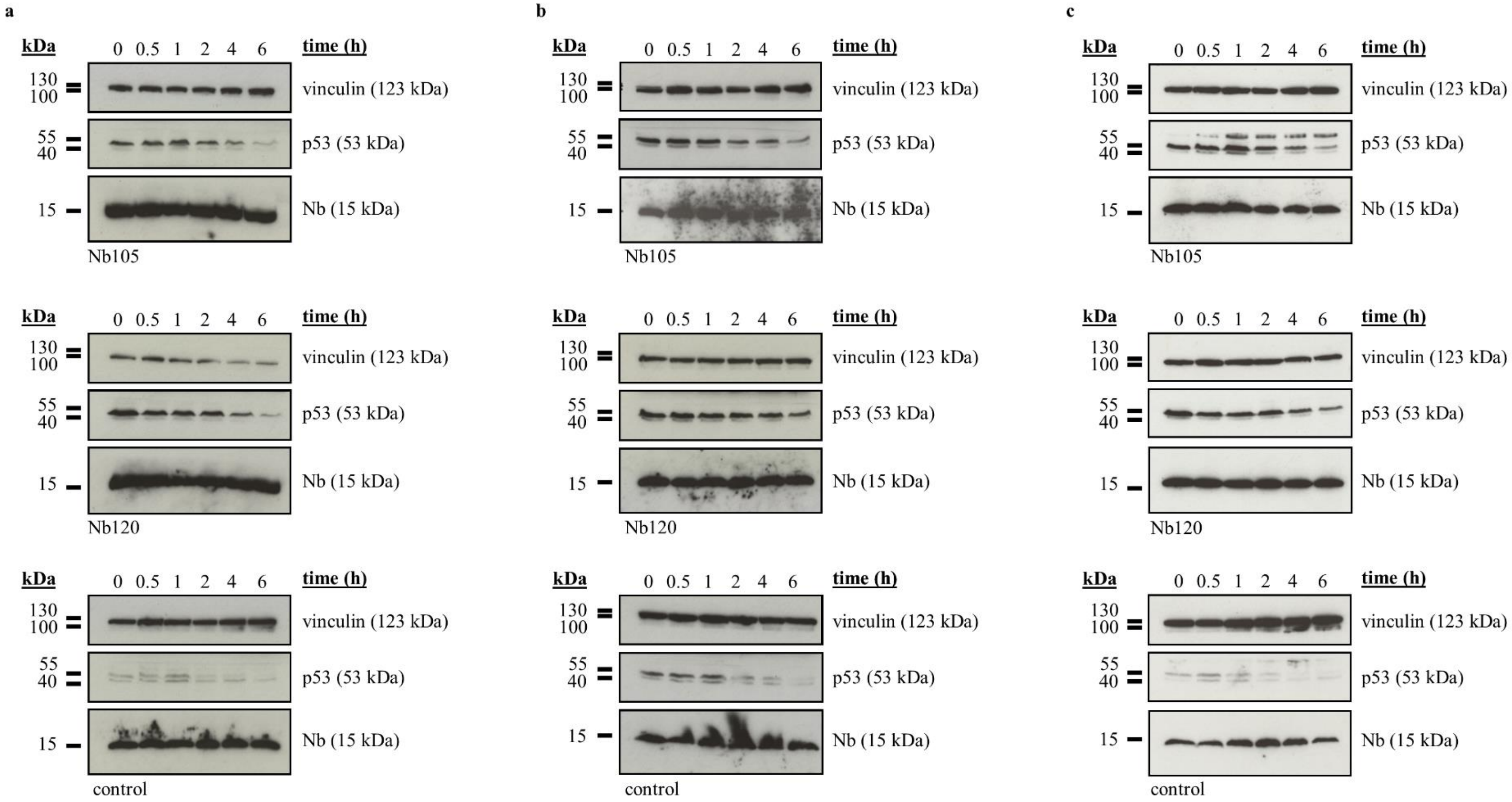

Supplementary Figure S3: **impact of the p53 DBD Nbs on p53 protein stability in HeLa cells.** Results of the additional repetitions of the CHX pulse chase assay performed in HeLa cells. HeLa cells transiently expressing FLAG-tagged p53 DBD Nb105, p53 DBD Nb120 or GFP Nb (negative control) were treated 24h post-transfection with 20 µg/ml CHX. Crude lysates (60 µg) were prepared at several time-points after CHX addition (i.e. 0h, 0.5h, 1h, 2h, 4h or 6h). The half-life of p53 was evaluated via western blot. Vinculin was implemented as loading control. The p53 degradation kinetics were substantially slower in HeLa cells expressing the p53 DBD Nbs. Significantly higher p53 levels were detected until 4h after CHX addition after intracellular expression of p53 DBD Nb120. Blots were cropped to the bands of interest. Samples derived from the same experiment and gels/blots were processed in parallel.

Supplementary Figure S4

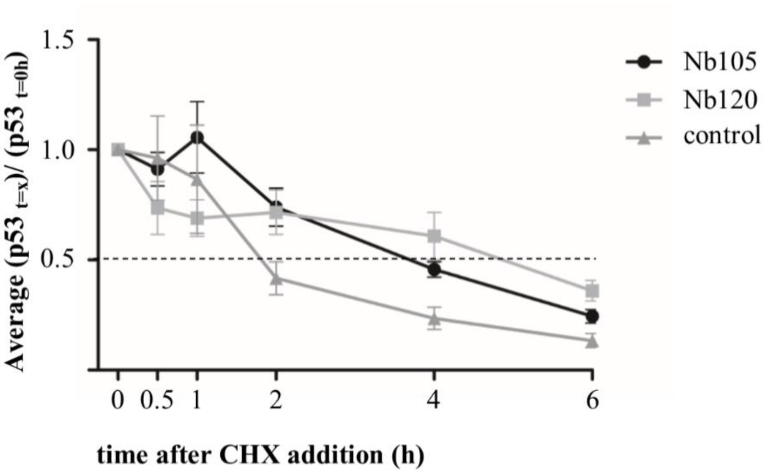

Supplementary Figure S4: the p53 DBD Nbs substantially prolong p53 half-life in HeLa cells. In order to determine the influence of the p53 DBD Nbs on p53 half-life more accurately, the data of the CHX pulse chase assay were re-analysed. In this analysis, the p53 levels that were measured for each condition at the start of the experiment (i.e. t = 0h) were considered as 100% and were used for an additional normalization of the data depicted in **Fig. 3**. The resulting values are depicted in the graph above (± SEM). The dotted line in the graph visualises when p53 levels have dropped to 50% of the initial levels. In HeLa cells that were transfected with the unrelated GFP Nb, p53 levels were halved ±2h after the addition of CHX. By contrast, a substantial increase in p53 half-life can be observed after intracellular expression of p53 DBD Nbs. The half-life of p53 increases to ± 4h and ± 5h in the presence of p53 DBD Nb105 and p53 DBD Nb120, respectively.

Supplementary Figure S5

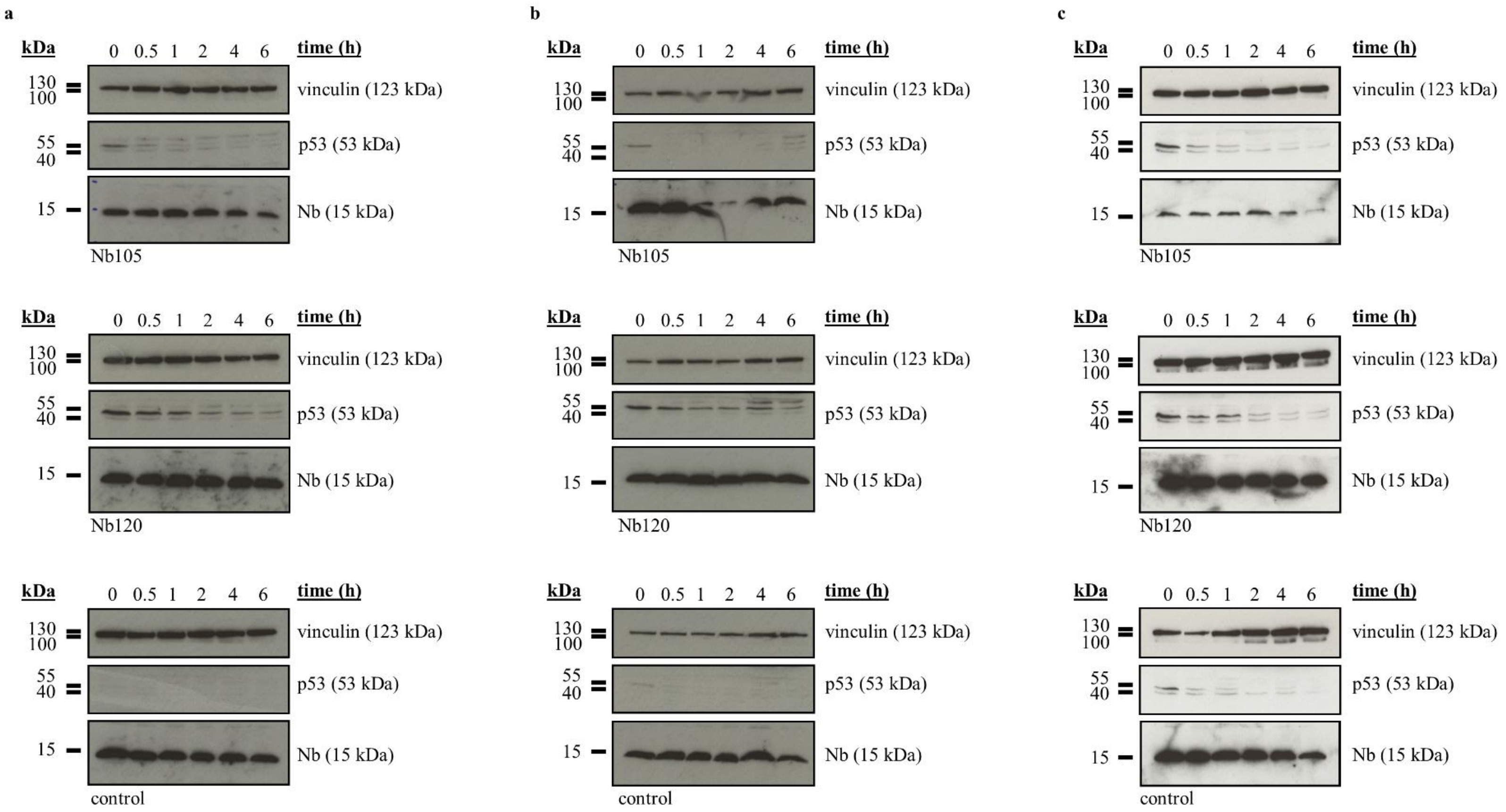

Supplementary Figure S5: p53 DBD Nbs have no significant impact on p53 protein stability in SiHa cells. Results of the additional repetitions of the CHX pulse chase assay performed in SiHa cells. SiHa cells transiently expressing FLAG-tagged p53 DBD Nb105, p53 DBD Nb120 or GFP Nb (negative control) were treated 24h post-transfection with 20 µg/ml CHX. Crude lysates (60 µg) were prepared at several time-points after CHX addition (i.e. 0h, 0.5h, 1h, 2h, 4h or 6h). The half-life of p53 was evaluated via western blot. Vinculin was implemented as loading control. Expression of the p53 DBD Nbs resulted in a significant augmentation of p53 levels at t = 0h, but this effect did not persist in time. A small increase in p53 levels was detected at several time points after CHX addition when p53 DBD Nb120 was intracellularly expressed, albeit these changes were not significant. Blots were cropped to the bands of interest. Samples derived from the same experiment and gels/blots were processed in parallel.

Supplementary Figure S6

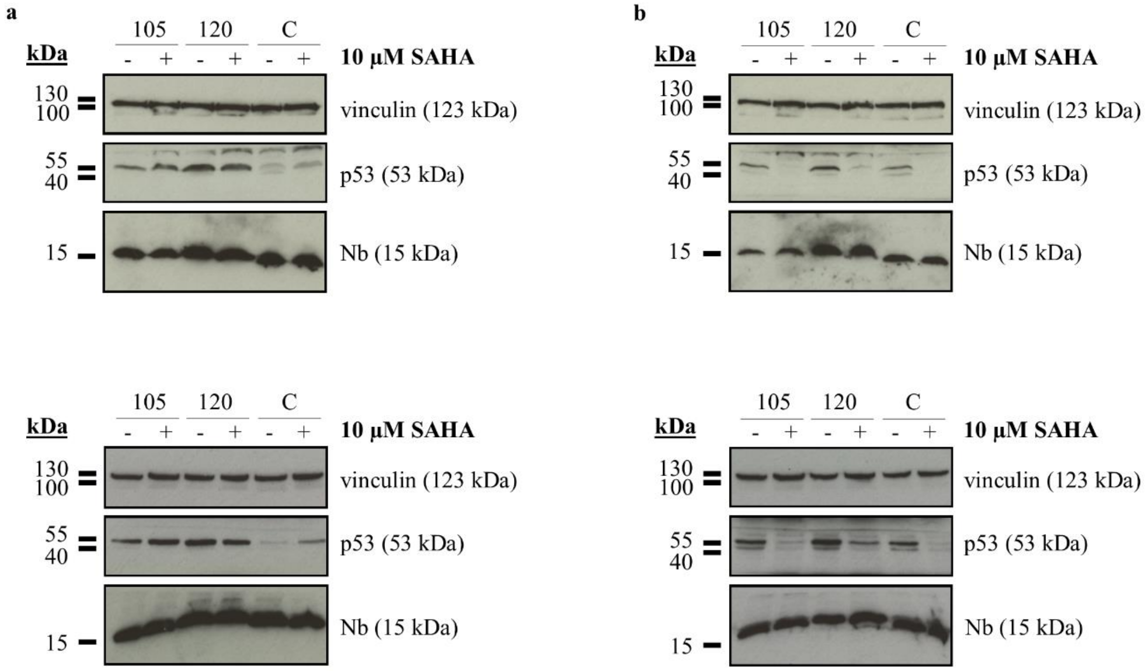

Supplementary Figure S6: **evaluation of p53 levels after co-treatment of HPV-infected cells with p53 DBD Nbs and the HDACi SAHA.** Western blot data of two additional repetitions of the experiment performed in HeLa **(a)** and SiHa **(b)** cells. Transfected cells received (or did not receive) 4h post-transfection an additional treatment with 10µM SAHA for a duration of 20h. A negative control was implemented where cells were transfected with the unrelated FLAG-tagged GFP Nb (C). Crude lysates (60 µg) were prepared 24h after transfection and p53 expression levels were analysed through western blotting. Vinculin served as a loading control. For reasons of clarity and conciseness, blots were cropped to the bands of interest.

Supplementary Figure S7

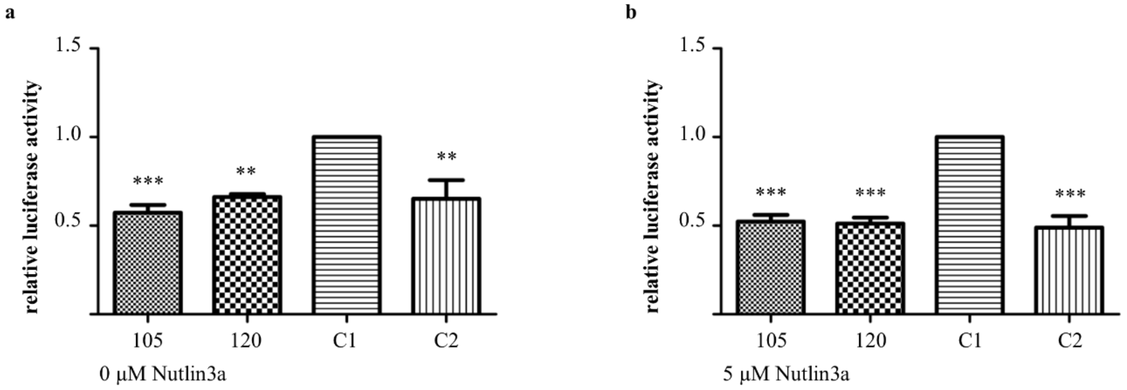

Supplementary Figure S7: the p53 DBD Nbs perturb the transcriptional program of p53 in U2OS pGL13 cells. It was investigated whether the p53 DBD Nbs exert an inhibitory effect over the functionality of p53 as a transcriptional transactivator in HPV-negative U2OS pGL13 cells. These cells express the pGL13 luciferase construct in a stable manner. Cells were transfected with the FLAG-tagged p53 DBD Nbs. A negative control was implemented whereby cells were transfected with the FLAG-tagged GFP Nb (C1). A positive control consisted of cells that transiently expressed the HA-tagged R175H p53 mutant, which exerts a dominant-negative effect over the activity of wild type p53 (C2). The transfected cells received (or did not receive) an additional treatment with 5 µM Nutlin3a for a duration of 20h. Values represent the mean luciferase activity relative to the negative control (±SEM) measured for triplicate samples in 4 independent experiments. Statistical analysis was performed via a one-way ANOVA, with Dunnett's multiple comparison test with a p-value < 0.05. **(a)** The p53 DBD Nbs inhibited the transactivation functions of p53 as effective as the R175H p53 mutant. Compared to the negative control (C1), the relative luciferase activity was reduced with 43% ($p < 0.001$) and 33 % ($p < 0.01$) under the influence of p53 DBD Nb105 and p53 DBD Nb120, respectively. In comparison, a reduction of 35% was observed after expression of the R175H p53 mutant (C2) ($p < 0.01$). **(b)** Similar observations were made after activation of p53 through the additional treatment of the transfected cells with 5 µM Nutlin3a. The relative luciferase activity dropped significantly with 48% (p53 DBD Nb105), 49% (p53 DBD Nb120) and 51% (R175H p53 mutant) ($p < 0.001$).

Supplementary Figure S8

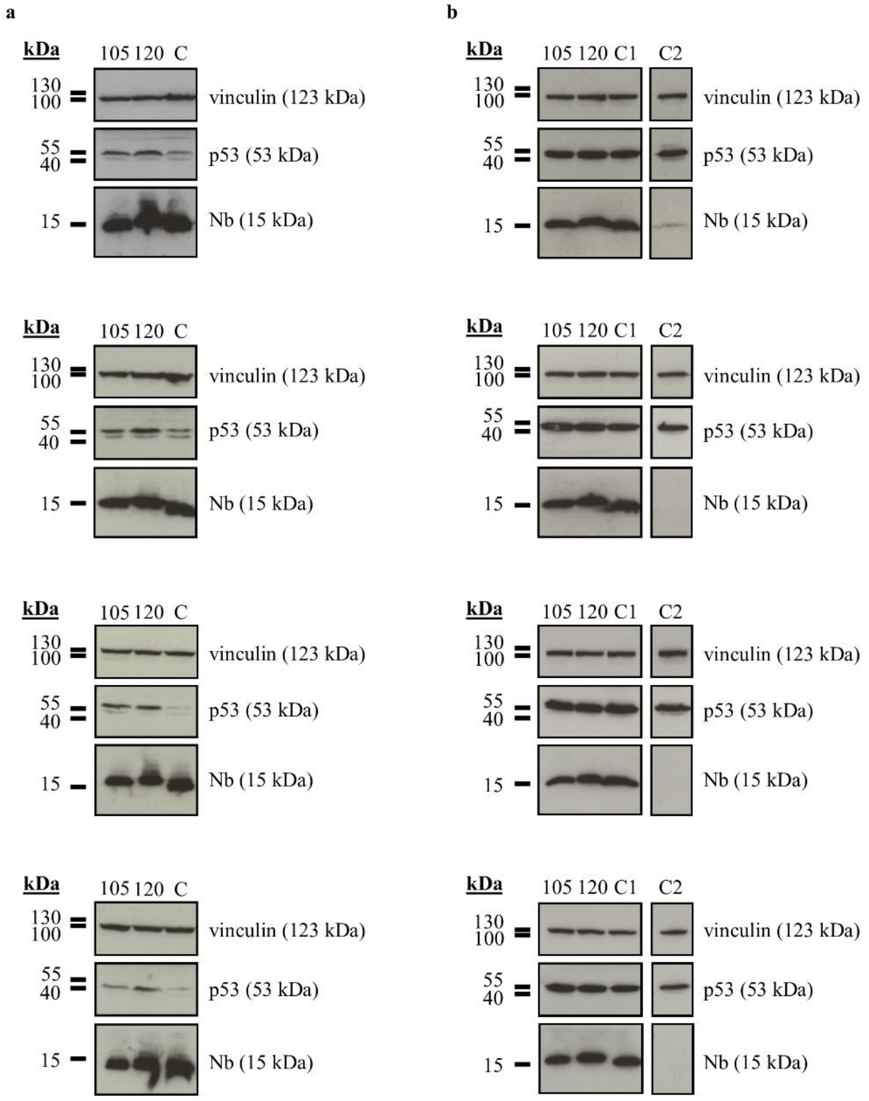

Supplementary Figure S8: evaluation of the transfection efficiency for each individual repeat of the transactivation assay, performed in HeLa cells and U2OS pGL13 cells. (a) HeLa cells were transfected with FLAG-tagged p53 DBD Nbs or an unrelated FLAG-tagged GFP Nb which was implemented as negative control (C**)**. **(b)** U2OS pGL13 cells were transfected with FLAG-tagged p53 DBD Nbs or an unrelated FLAG-tagged GFP Nb, which served as negative control (C1). Here, an additional positive control was included whereby cells were transfected with HA-tagged R175H p53 mutant (C2). After re-seeding the transfected cells into 96-well plates, crude lysates were prepared from the remainder cells. Samples of crude lysate (HeLa cells: 60 µg, U2OS pGL13 cells: 40 µg) were loaded onto a 15% SDS gel and analysis of protein expression levels was performed via western blotting. Vinculin served as a loading control. For reasons of clarity and conciseness, blots were cropped to the bands of interest. A full-length blot is shown in **Supplementary Fig. S13**.

Supplementary Figure S9

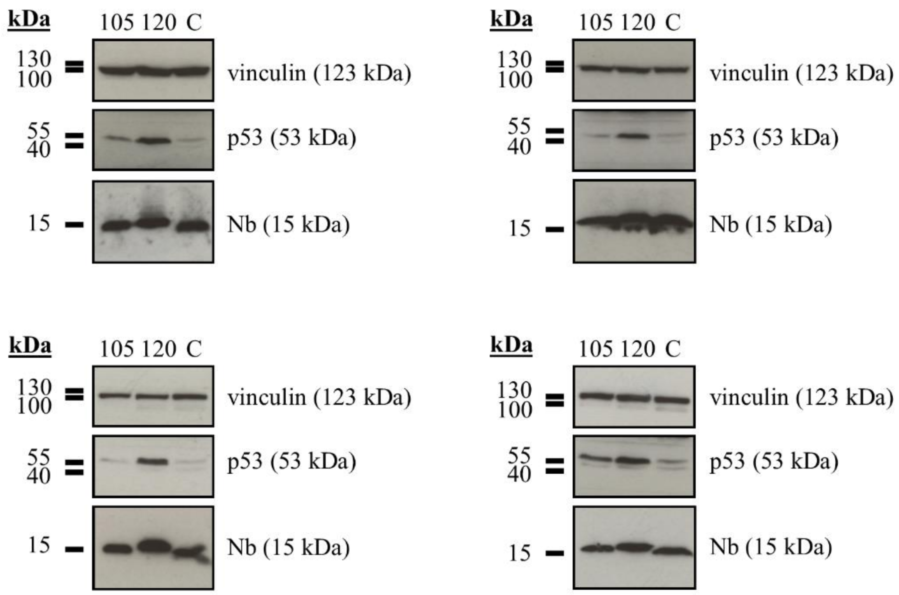

Supplementary Figure S9: evaluation of the transfection efficiency for each individual repeat of the XTT assay performed in HeLa cells. HeLa cells were transfected with FLAG-tagged p53 DBD Nbs or an unrelated FLAG-tagged GFP Nb which was implemented as negative control (C). After re-seeding the transfected cells into 96-well plates, crude lysates (60 µg) were prepared from the remainder cells. Analysis of protein expression levels was performed via western blotting. Vinculin served as a loading control. For reasons of clarity and conciseness, blots were cropped to the bands of interest. A full-length blot is shown in **Supplementary Fig. S13**.

Supplementary Figure S10

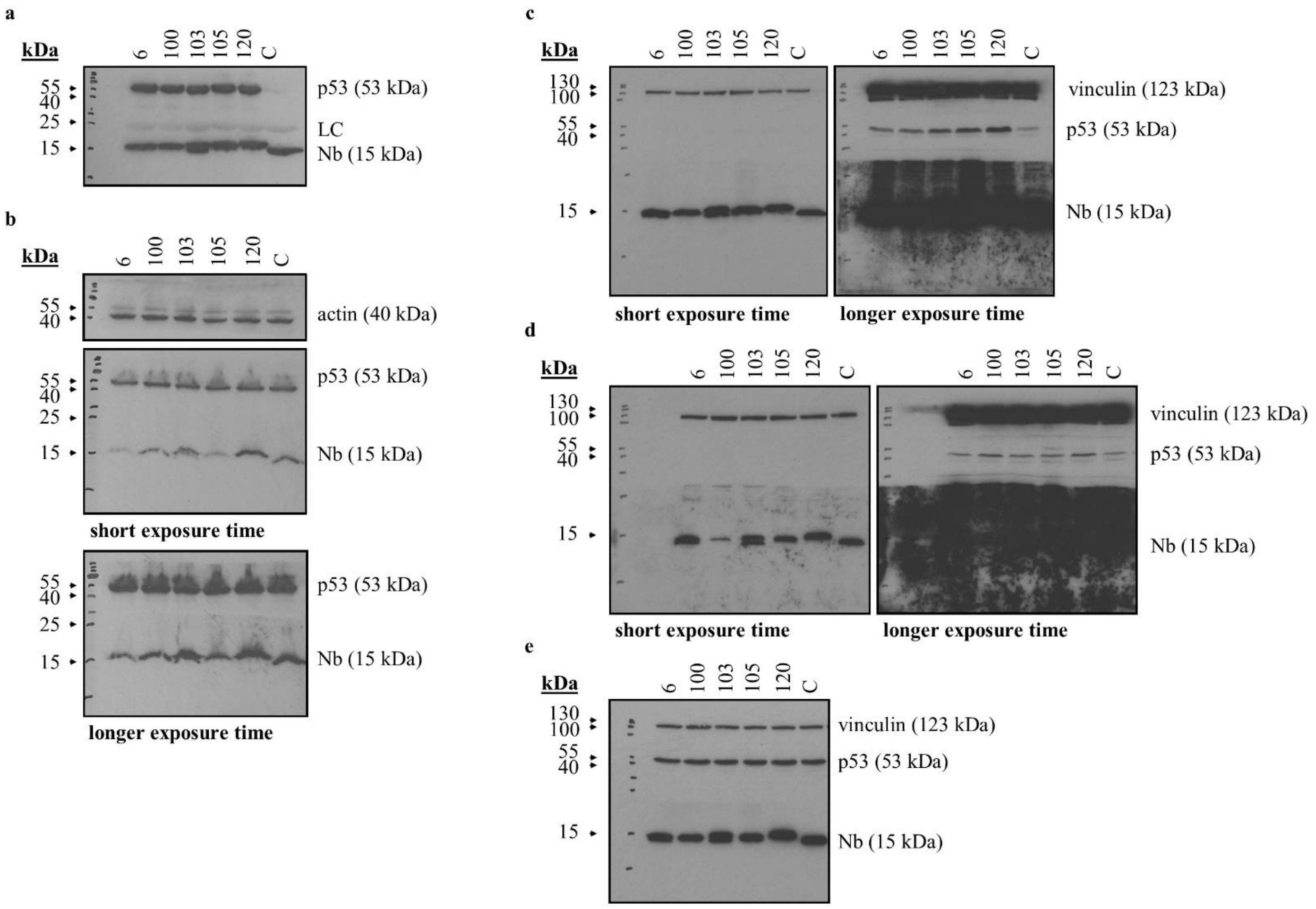

Supplementary Figure S10: full–length blots of the experiments shown in **(a)** Fig. 1a, **(b)** Fig. 1b, **(c)** Fig. 2a, **(d)** Fig. 2b and **(e)** Fig. 2c. Uncropped images are labelled as in the main text.

Supplementary Figure S11

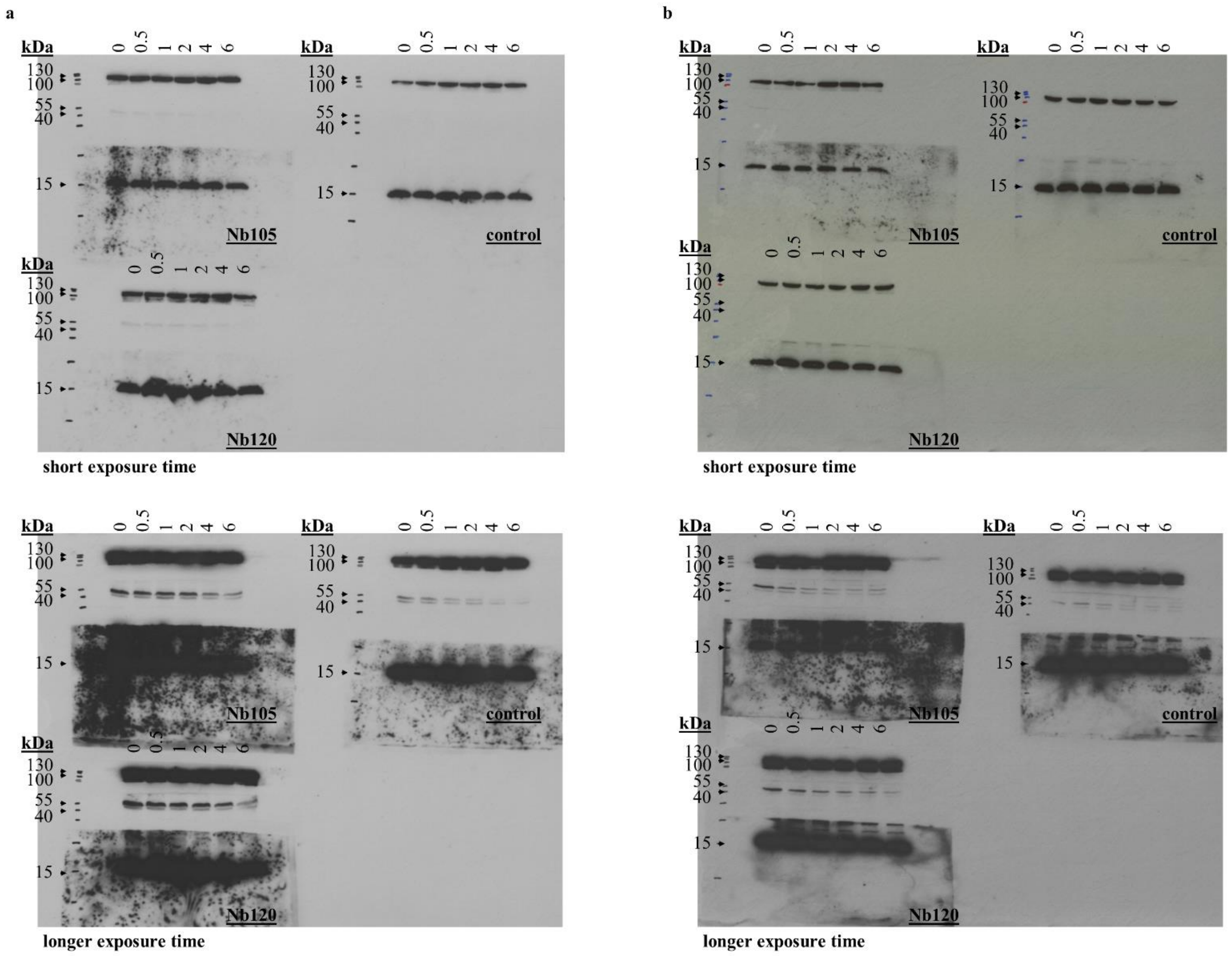

Supplementary Figure S11: full–length blots of the experiments shown in **(a)** Fig. 3a and **(b)** Fig. 3b. Uncropped images are labelled as in the main text.

Supplementary Figure S12

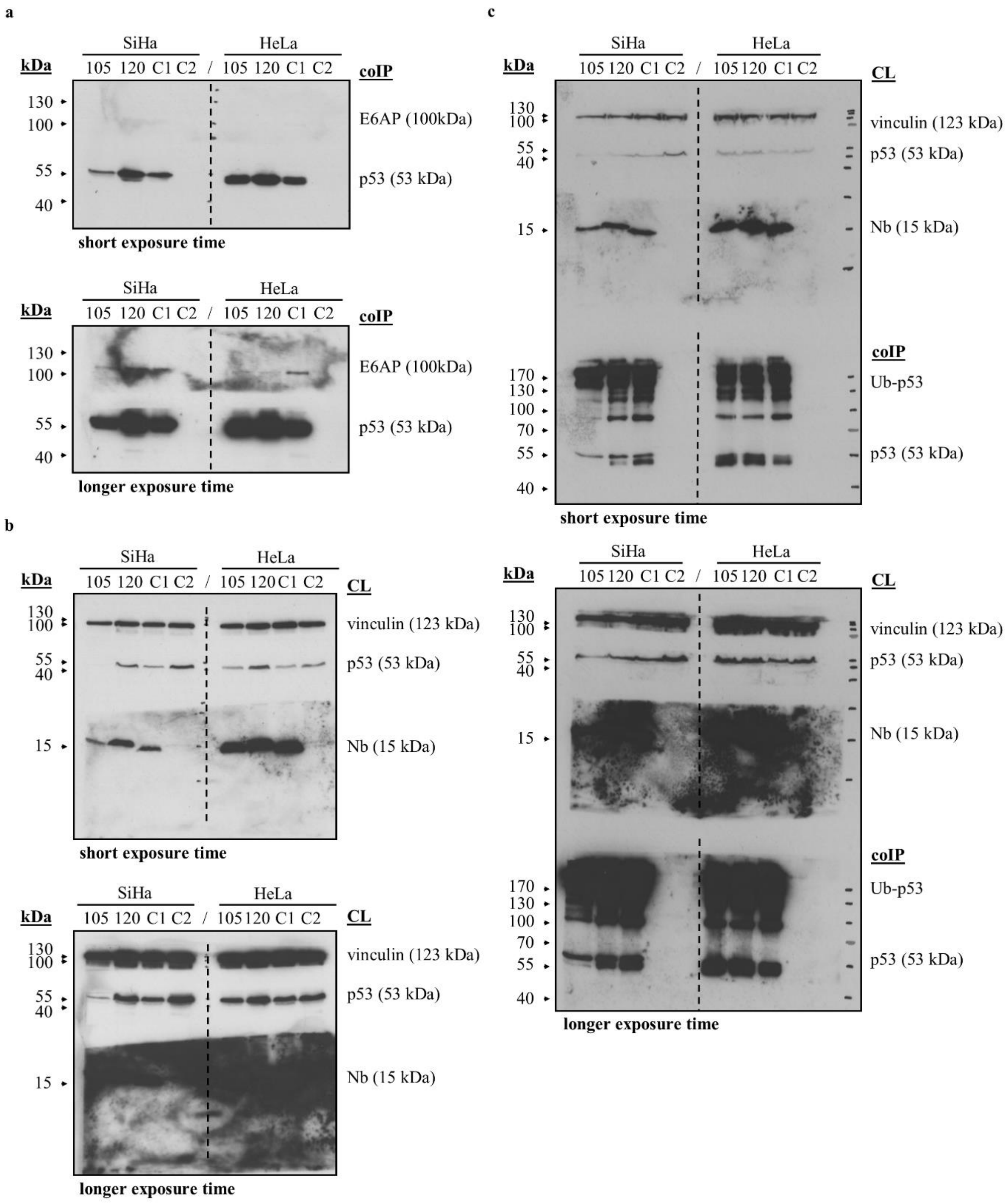

Supplementary Figure S12: full–length blots of the experiments shown in **(a)** Fig. 4a (coIP), **(b)** Fig. 4a (CL) and **(c)** Fig. 4b. Uncropped images are labelled as in the main text.

Supplementary Figure S13

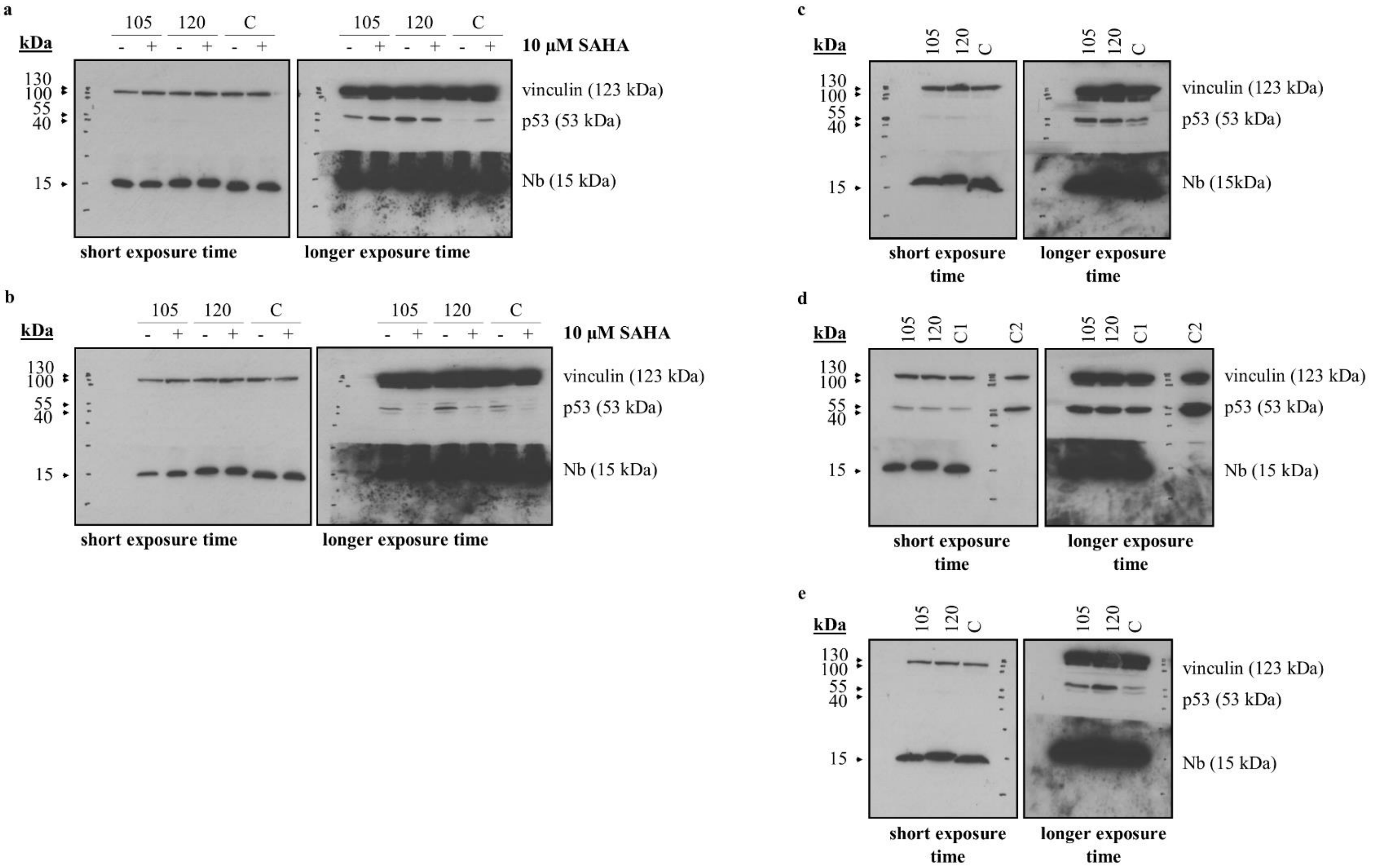

Supplementary Figure S13: full–length blots of the experiments shown in **(a)** Fig. 5a, **(b)** Fig. 5b, **(c)** Supplementary Fig. S8a, **(d)** Supplementary Fig. S8b and **(e)** Supplementary Fig. S9. Uncropped images are labelled as in the main text.

Supplementary Methods

Antibodies and reagents

Anti-FLAG M2 affinity gel (A2220), mouse monoclonal anti-p53 (DO-1, P6874), mouse monoclonal anti-vinculin (clone hVIN-1, V9131), rabbit polyclonal anti-FLAG (F7425), DAPI (D8417), nutlin-3a (N6287), staurosporine (S4400), cycloheximide (c1988) and N-ethylmaleimide (E3876) were purchased from Sigma-Aldrich. Alexa Fluor 488/594 goat anti-rabbit or mouse (A11034, A11032) and Clean-Blot IP detection reagent (HRP) (21230) were purchased from Thermo Fisher Scientific. The XTT cell proliferation kit II (11465015001) and mouse monoclonal anti-HA (clone 12CA5, 11583816001) were purchased from Roche. Rabbit monoclonal anti-UBE3A (ab126765), goat polyclonal anti-beta actin (ab8229) and rabbit anti-goat IgG H&L (HRP) (ab6741) were purchased from Abcam. ECL anti-mouse or rabbit IgG HRP-linked substrates (NA931, NA9340) were purchased from GE Healthcare. JetPrime transfection reagent (114-15) was purchased from Polyplus Transfection. SAHA (vorinostat) (S1047) was purchased from Selleck Chemicals.

Generation of p53 DBD Nbs

Nbs targeted against the DNA-binding domain of p53 (p53 DBD, AA 92-312) were generated in collaboration with the VIB Nanobody Service Facility as previously described.[1] In short, an alpaca was injected subcutaneously with 200 µg p53 DBD (fused to His_6-tag) on days 0, 7, 14, 21, 28 and 35. At day 39, lymphocytes were prepared from the collected anticoagulated blood. Thereafter, a VHH library was constructed and screened to single out antigen-specific Nbs. The VHH library contained 1 x 10^8 independent transformants, of which 90% harbored the vector with the right insert size. Subsequently, 4 consecutive rounds of panning were performed on the solid-phase coated antigen during which enrichment for antigen-specific phages was detected after the 2nd, 3rd and 4th round of panning. A total of 380 individual colonies were randomly selected and analyzed by ELISA for the presence of antigen-specific Nbs in the periplasmic extracts. Positive colonies were analysed by nucleotide sequencing

cDNA cloning

The expression plasmid pHEN4 was used to subclone the Nbs into the mammalian expression vector pMET7-FLAG. Subcloning of the Nbs was performed using the Cold Fusion™ cloning kit (System Biosciences). Following primers were used to subclone the Nbs into the pMET7-FLAG vector: 5' GAT GAC GAC GAT AAG GAA TTC CAG GTG CAG CTG CAG GAG 3' (forward primer) and 5' GGT TTT TTC TCT AGA GCG GCC GCT CAG CTG GAG ACG GTG ACC T 3' (reverse primer).

Supplementary references

1. Van den Abbeele, A. *et al.* A llama-derived gelsolin single-domain antibody blocks gelsolin-G-actin interaction. *Cell. Mol. Life Sci.* **67,** 1519-1535, doi:10.1007/s00018-010-0266-1 (2010).
